# The effect of brain tissue anisotropy on the electric field caused by transcranial electric stimulation: Sensitivity analysis and magnetic resonance electrical impedance tomography

**DOI:** 10.1162/imag_a_00481

**Published:** 2025-02-26

**Authors:** Mohsen Mosayebi-Samani, Teresa Cunha, Hasan Hüseyin Eroğlu, Hartwig Roman Siebner, Michael A. Nitsche, Axel Thielscher

**Affiliations:** Department of Psychology and Neurosciences, Leibniz Research Center for Working Environment and Human Factors, Dortmund, Germany; Section for Magnetic Resonance, DTU Health Tech, Technical University of Denmark, Kgs Lyngby, Denmark; Danish Research Centre for Magnetic Resonance, Centre for Functional and Diagnostic Imaging and Research, Copenhagen University Hospital—Amager and Hvidovre, Copenhagen, Denmark; Department of Neurology, Copenhagen University Hospital Bispebjerg, Copenhagen, Denmark; Institute for Clinical Medicine, Faculty of Medical and Health Sciences, University of Copenhagen, Copenhagen, Denmark; Bielefeld University, University Hospital OWL, Protestant Hospital of Bethel Foundation, University Clinic of Psychiatry and Psychotherapy, Bielefeld, Germany; German Centre for Mental Health (DZPG), Bochum, Germany

**Keywords:** magnetic resonance electrical impedance tomography (MREIT), Magnetic resonance current density imaging (MRCDI), volume conductor modeling, transcranial electric stimulation, tissue conductivity

## Abstract

Calculations of the electric field (**E**-field) are important for addressing the variability in the physical dose of transcranial electric stimulation (tES). These calculations rely on precise knowledge of the individual head and brain anatomy and on choosing the appropriate ohmic conductivities for the different tissue compartments. In particular, the conductivity of brain white matter and to a lesser extent gray matter is anisotropic. Consensus on the importance to account for the conductivity anisotropy of the brain in the**E**-field calculations is still lacking. Most simulation studies use isotropic conductivities, which avoids the need for diffusion tensor imaging (DTI) data and lowers practical complexity. On the other hand, in magnetic resonance electrical impedance tomography (MREIT) that employs measurements of the tiny magnetic fields caused by the tES-induced current flow, diffusion anisotropy measured by DTI has been put forward as a key parameter for the reconstruction of the brain conductivity at the individual level. Here, we conducted a series of three sub-studies to systematically assess the effect of brain anisotropy on the tES-induced**E**-field in cortical gray matter and to compare in-vivo MREIT data with simulated data from isotropic and anisotropic head models. In sub-study 1, we employed simulations to demonstrate that sparse knowledge of the ohmic tissue conductivities is the main source of uncertainty, while the modeling of brain anisotropy has comparatively small effects on the simulated**E**-field. In sub-study 2, we compared simulations with in-vivo MREIT data and found that optimizing the conductivities of the modeled tissue compartments enhanced the agreement between simulated and measured data. Modeling brain conductivity as anisotropic had no impact on this optimization process. In sub-study 3, we used simulations to test how the differences in the tES-induced current flow caused by isotropic versus anisotropic brain conductivities affect the results of the “DT-MREIT” algorithm, which enables voxel-wise reconstructions of brain tissue conductivity. The algorithm performed similarly in both cases. On the other hand, the results were worse in a more realistic scenario where the reconstruction was based on simulated MREIT data (rather than simulated current densities). Together, our findings underscore the relevance of an accurate knowledge of the tissue conductivities for calculations of the tES-induced**E**-field. When cortical gray matter is the target for tES, modeling brain conductivity as anisotropic based on DTI data does not add substantial benefit. While in-vivo MREIT data generally show promise for refining the conductivity estimates of biological tissue at low frequencies, MREIT appears to be only weakly sensitive to the conductivity anisotropy of brain tissue.

## 1 Introduction

Transcranial electrical brain stimulation (tES) non-invasively modulates cortical neural activity, making it a valuable tool to study human brain function. It might also be useful as a treatment of neurological or psychiatric disorders (Flöel, 2014;Lefaucheur et al., 2017;Polanía et al., 2018), but its efficacy is still hampered by a large inter-individual variability of effects. Mechanistically, tES likely alters neural activity by shifting cell membrane potentials, so that its effects depend on the properties of the electrical field induced in the cortical sheet, including its strength and local direction (Alekseichuk et al., 2019,^2022^;Miranda et al., 2013;Tran et al., 2022;Yavari et al., 2018).

Simulation studies consistently show that the tES-induced electric field is strongly affected by individual head and brain anatomy, indicating that electric field differences contribute to the variability of the physiological stimulation effects (Kasten et al., 2019;Laakso et al., 2015;Mosayebi-Samani et al., 2021;G. B. Saturnino, Thielscher, et al., 2019). Conversely, simulation procedures could be used to individually adapt the delivered tES dosage to result in uniform received dosages over individuals. The accuracy of the simulated field, which is critical for application of simulation approaches, however, depends on the anatomical accuracy of the underlying volume conductor model of the head (Datta et al., 2009;Laakso et al., 2015;Miranda et al., 2013;Puonti, Saturnino, et al., 2020) and the accuracy of the ohmic conductivity values assigned to the modeled tissue compartments (Antonakakis et al., 2020;McCann et al., 2019;G. B. Saturnino, Thielscher, et al., 2019). Unfortunately, conductivity measurements so far require invasive procedures and the values reported in the existing sparse literature show a large variability, rendering also the calculated electric fields uncertain (G. B. Saturnino, Thielscher, et al., 2019). This might also limit the usefulness of electric field simulations for controlling and optimizing the individually applied electric field dose.

The conductivity of brain tissue is anisotropic at low frequencies (<100 kHz), as the membranes of the spatially aligned neural fibers restrict the ionic current flow (Tuch et al., 2001). In particular, the neural fibers in the white matter exhibit location-dependent preferential orientations, which result in higher ohmic conductivities for current directions parallel to the fibers compared to orthogonal current directions. Simulation studies estimating the conductivity anisotropy of brain tissue from MR diffusion tensor imaging (DTI) suggest that anisotropy moderately influences the tES-induced electric field in the cortical gray matter where the physiological stimulation effects are thought to occur (Opitz et al., 2015;Rampersad et al., 2014;Suh et al., 2012). However, several alternative conversion schemes between DTI and conductivity anisotropy have been proposed (Güllmar et al., 2010;Rullmann et al., 2009;Tuch et al., 2001), and the strength of the influence of conductivity anisotropy on the electric field varies with the conversion scheme (Opitz et al., 2015). The accuracy of the different conversion schemes is unclear so far due to lacking ground truth data. Approaches that combine DTI data with MR-based measurements of the tiny magnetic fields caused by the tES-induced current flow (termed MR Electrical Impedance Tomography—MREIT—or MR Current Density Imaging—MRCDI;Goksu, Hanson, et al., 2018;Scott et al., 1992;Woo & Seo, 2008) have been suggested to improve the accuracy of the reconstructed conductivity distribution of the brain (Chauhan et al., 2018;Kwon et al., 2014). One prevalent algorithm, termed DT-MREIT (Kwon et al., 2014), in principle, enables conductivity estimates on a single voxel level and might be a valuable way to improve the accuracy of head volume conductor models if it reaches sufficient accuracy for human in-vivo MR data. However, while the algorithm has been shown to work well in phantoms with simplified geometries, the approach lacks stringent validation for human brain data. In sum, it remains unclear to what extent deriving estimates of the anisotropic conductivity of the brain from MR data improves the accuracy of the calculated electric fields.

This study has three aims: First, we aim to quantify how incorporating estimations of the brain conductivity anisotropy from DTI into the head volume conductor model changes the simulated electric field in gray matter, compared to the uncertainty ranges of the**E**-fields caused by incomplete knowledge of tissue conductivities. While previous studies have addressed some of these aspects in isolation (Indahlastari et al., 2020;Schmidt & van Rienen, 2012), we here aim to directly compare their relative impact on the**E**-field simulations. In particular, we aim to compare the size of this effect with the uncertainty of the electric field that is caused by insufficient knowledge of the tissue conductivities in first instance. Second, using MREIT data, we wish to test whether including brain conductivity anisotropy estimated from DTI data into the calculations improves the fit between the simulated and measured current-induced magnetic fields, compared to the use of isotropic and homogeneous conductivities. Third, we wish to determine whether the outcome of the DT-MREIT algorithm (Kwon et al., 2014) is sensitive to the differences in the received current densities that are related to anisotropic versus isotropic and homogeneous brain conductivities. In summary, our study will enable us to conclude whether the anisotropy of brain conductivity has a relevant influence on the tES current flow in the human brain, and whether it can be faithfully estimated from MR data.

## 2 Material and Methods

Sections 2.1to2.4of this article cover general methodological aspects that apply to all three sub-studies. This is followed by specific information for the sub-studies inSections 2.5to2.7.

### 2.1 Participants

Five volunteers (four male) were recruited for this study. All participants participated in both the first and second sub-studies, whereas the openly accessible head model ‘ernie’ was used for the third sub-study (G. Saturnino et al., 2015). The participants had no previous or present neurological or psychiatric disorders. Written informed consent was obtained from the participants prior to the scans and they were screened for contraindications to MRI and tES. The study complied with the Helsinki declaration on human experimentation and was approved by the Ethics Committee of the Capital Region of Denmark. In addition, the analyses of sub-study 1 were repeated in 20 additional subjects (10 male) which were randomly selected from the Human Connectome Project (HCP) dataset.

### 2.2 MR imaging and preprocessing

A 3T MRI scanner equipped with a 64-channel head coil (Magnetom PRISMA, Siemens) was used to acquire the MR data, including structural and diffusion MR images, and MREIT data.

**Fig. 1.**
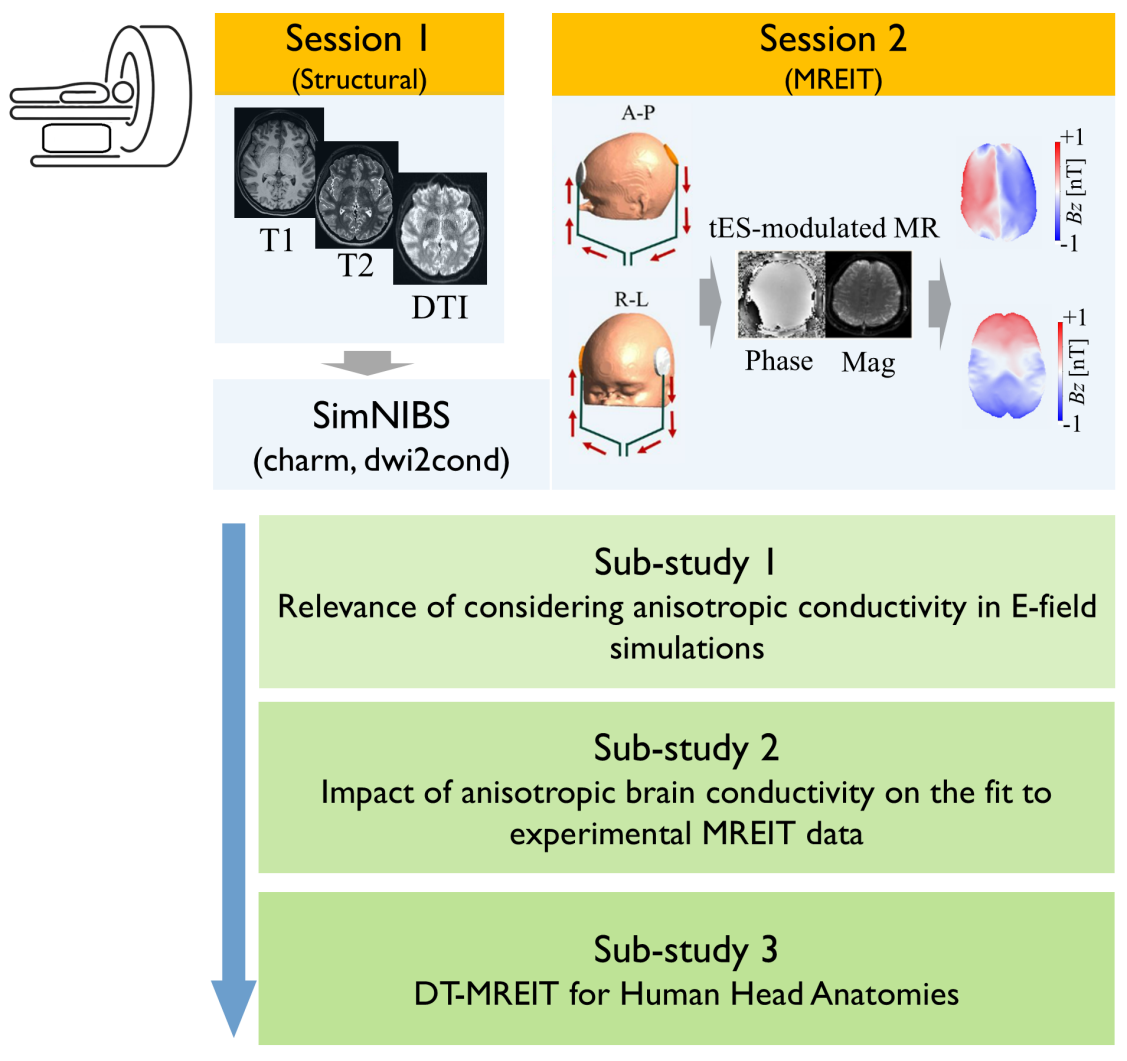
Course of the study. Five volunteers participated in two consecutive MR data acquisition sessions, which included recording of structural and diffusion MR images (session 1) and of MREIT data (A-P: anterior-posterior montage and R-L: right-lift montage; session 2). The acquired MR data were utilized in sub-studies 1 and 2 to systematically test the significance of integrating brain conductivity anisotropy in tES field simulations and for comparisons of those simulations with MREIT data. The first sub-study used simulations to test the impact of brain conductivity anisotropy on the tES-induced electric fields and compare it to the general uncertainty of the electric fields due to insufficient knowledge of the tissue conductivities. The second sub-study assessed whether incorporating estimates of brain conductivity anisotropy in the calculations improved the match between simulated and measured MREIT data. Finally, sub-study 3 evaluated how the performance of the DT-MREIT algorithm in reconstructing the conductivity of brain tissue depends on the accuracy of the supplied current density data. The latter was simulated using anisotropic brain conductivities (ground truth), simulated using matched isotropic brain conductivities, or reconstructed from simulated MREIT data.

- **T1- and T2-weighted**structural images were acquired for automatic reconstruction of volume conductor models of the head (details are covered below). The T1-weighted structural images were based on a Magnetization-Prepared Rapid Acquisition Gradient-Echo (MPRAGE) sequence with a number of slices N_sli_= 208, image matrix 256 x 256, a voxel size of 1 x 1 x 1 mm^3^, a tip angle α = 9˚, TR = 2,700 ms, TE = 3.63 ms, and an inversion time of TI = 1,090 ms with selective water excitation. The T2-weighted structural images used a Sampling Perfection with Application-optimized Contrasts using a different flip-angle Evolutions (SPACE) sequence with N_sli_= 208, image matrix 256 x 256, voxel size 1 x 1 x 1 mm^3^, TR = 3,200 ms, TE = 408 ms, and turbo factor 282.
- **Diffusion MRI**was acquired to determine water diffusion tensors in gray and white matter, from which the conductivity anisotropy tensors were estimated in a post-processing step. A twice-refocused SE-EPI sequence was used with 72 axial slices, matrix size = 128 × 128, voxel size 1.9 × 1.9 × 2.1 mm^3^, TR = 10,500 ms, TE = 105 ms, 6/8 phase partial Fourier, GRAPPA acceleration factor 2, and 7 averages. Thirty diffusion directions were acquired with a b-value of 1,000 s/mm^2^as well as five interspersed b = 0 s/mm^2^images (Opitz et al., 2011;Windhoff et al., 2013). The raw diffusion MR images were automatically preprocessed based on FSL tools using the SimNIBS script*dwi2cond*, which uses FSL topup and eddy for correction of static distortions and eddy-current artefacts (Andersson et al., 2003;Smith et al., 2004), and FSL dtifit to estimate water diffusion tensors from the diffusion MR data. The diffusion tensors were spatially coregistered to the T1-weighted structural scan for further use using a rigid transformation.
- The tES-current-induced magnetic field changes were measured using an in-house developed**MREIT**acquisition method (Göksu et al., 2021). The multi-gradient-echo MREIT sequence has an FOV = 224 × 183 mm, α = 30°,*TE*= [5.6, 14.4, 23.2, 32, 40.8, 49.6, 58.4, 67.2] ms, TR = 80 ms, and combines gradient spoiling (*φ*_*sp*_= 16π) and RF spoiling with an acquisition-weighting scheme to ensure robustness to physiological noise and an SNR-efficient sampling of the tES-current-induced phase changes of the MR signal (Goksu, Hanson, et al., 2018;Goksu, Scheffler, et al., 2018). Rectangular tES current waveforms were created using a waveform generator (Agilent Technologies, CA, USA) and applied in synchrony with the MREIT pulse sequence. They were amplified to ±1 mA magnitude using a battery-powered MR-compatible electrical stimulator (neuroConn GmbH, Ilmenau, Germany) and finally applied over the scalp through two sets of custom-made circular rubber electrodes (5 cm in diameter) (Gregersen et al., 2021), in the right-left (R-L) and anterior-posterior (A-P) directions (Fig. 1). A single slice covering the bottom part of the brain was measured for both directions. The tES current-induced magnetic fields were calculated from the measured MR phase data as detailed inGoksu, Hanson, et al. (2018). Note that the MREIT approach is only sensitive to current-induced magnetic fields parallel to the direction of the static magnetic field (i.e., to the z-component of the magnetic flux density, termed*B_z_*in the following).
- The measured MREIT data are not only influenced by the magnetic field caused by the tES current flow inside the head, but also by the field caused by the currents flow in the cables connecting the stimulator to the scalp electrodes. In order to correct for this undesired influence, the cable paths and exact positions of the rubber stimulation electrodes were delineated in a high-resolution structural image based on the Pointwise Encoding Time reduction with Radial Acquisition (PETRA) sequence with number of slices = 320, image matrix = 320 x 320, voxel size 0.9 x 0.9 x 0.9 mm^3^, tip angle α = 6˚, TR = 3.61 ms, TE = 0.07 ms, inversion time = 0.5 s, BW = 359 Hz/pixel, and turbo factor 400 (Göksu et al., 2019). The unwanted effects of the magnetic stray field, caused by the cable currents on the*B_z_*measurements, could then be corrected using a straight-forward procedure that relies on the Biot-Savart law (Göksu et al., 2019). The stray-field-corrected*B_z_*data were then used in the remainder of the study.

### 2.3 Estimation of anisotropic brain conductivity from DTI data

We used two different approaches to map the water diffusion tensors measured by DTI to conductivity tensors, 1) ‘direct mapping’ (Rullmann et al., 2009;Tuch et al., 2001) and 2) ‘volume normalized’ (Güllmar et al., 2010). The first approach assumes a linear relationship between the eigenvalues of the diffusion and conductivity tensors

$$\sigma_{i}=sd_{i}$$ (1)

where$\sigma_{i}$and$d_{i}$denote the i-th (i∈{1,2,3}) conductivity and diffusion eigenvalues, respectively, and*s*denotes a scaling factor (Tuch et al., 2001). Here, the scaling factor*s*was selected such that the geometric mean of the conductivity eigenvalues, averaged across voxels in the brain, fitted that of the isotropic conductivities as reported in the literature. Thereby, a single factor*s*was chosen for GM and WM that matched the mean conductivities derived from DTI for both tissue types as well as possible in a least-squares sense to the isotropic reference values (Rullmann et al., 2009):

$$s=\;\frac{s_{WM}\sigma_{WM}^{iso}+s_{GM}\sigma_{GM}^{iso}}{s_{WM}^{2}+s_{GM}^{2}}$$ (2)

$$s_{WM/GM}=\;\sqrt[{3}]{\frac{\sum_{i=1}^{\text{N}}d_{1}.\;d_{2}.d_{3}}{\text{N}}}$$ (3)

where N denotes the number of voxels in GM and WM, and$\sigma_{WM/GM}^{iso}$denotes the isotropic conductivities of WM and GM, respectively. The isotropic conductivities were set depending on the goals of the sub-studies, as described inSections 2.5to2.7.

While the direct mapping approach ensures that the conductivities are on average in a reasonable range, an alternative ‘volume normalized’ approach is to locally match the geometric mean of the conductivity eigenvalues of each single voxel to that of an isotropic reference value (Güllmar et al., 2010). This approach matches the volume of the ellipsoid defined by the conductivity tensor to that of a corresponding isotropic conductivity sphere, with the adjusted conductivity eigenvalues being determined by:

$$\sigma_{i}=\;\frac{d_{i}}{\sqrt[{3}]{d_{1}.\;d_{2}.\;d_{3}}}\;\;.\;\;\sigma_{WM/GM}^{iso}$$ (4)

### 2.4 Forward simulations of the current flow and the current-induced magnetic field

The calculations of the electric fields (**E**-fields) were conducted using the Finite Element Method (FEM) implemented in SimNIBS 4.0.1 (Puonti, Van Leemput, et al., 2020;G. B. Saturnino, Madsen, et al., 2019;Thielscher et al., 2015). For each participant, an anatomically realistic volume conductor model of the head was automatically created from the structural T1- and T2-weighted MR images using the*charm*pipeline of SimNIBS. The model consists of 9 tissue compartments (gray matter (GM), white matter (WM), cerebrospinal fluid (CSF), scalp, compact bone of the skull, spongy bone of the skull, eyes, veins and arteries (“blood”), and rectus muscles),Table 1. The gray matter central surfaces (i.e., surfaces placed in gray matter halfway between the pial surface and the gray-white matter boundary) of the two hemispheres of the cerebrum were reconstructed using CAT12 (Gaser et al., 2022) functionalities embedded into charm, for subsequent use in the field simulations. The positions of the simulated scalp electrodes were determined according to the 10–20 EEG standard for sub-study 1 (electrode positions AFz and POz for the A-P montage, and positions C5 and C6 for an R-L montage) and from the PETRA images for sub-studies 2 and 3. In addition, in sub-study 1, the conventional left-M1 tES montage (M1-SO) with the target electrode at C3 and the return electrode above the supraorbital area (SO; electrode center at Fp2) was tested. For the used ohmic conductivities of tissue elements and electrode configurations, seeSections 2.5to2.7. The**E**-fields and current densities were calculated using the FEM in SimNIBS for a tES current strength of 1 mA baseline-to-peak. In addition, the z-component of the tES-current-induced magnetic fields (*B_z_*-fields) was determined from the current densities by applying the Biot–Savart law using the Fast Fourier Transform (Yazdanian et al., 2020). For further analyses, the**E**-fields were extracted at the positions of the gray matter central surfaces of the two hemispheres, in line with the hypothesis that the physiological effects of tES are caused by modulation of neural activity in the cortical sheet. The*B_z_*-fields were extracted in axial 2D slices and masks were used to only maintain*B_z_*data in GM, WM, and CSF, corresponding to the characteristics of the measured MREIT data.

**Table 1.** Ohmic conductivities for the tissue compartments of the head (column 2) and limits of the electrical conductivities assigned to the different tissue types in the generalized polynomial chaos (gPC) model (column 3).

Tissues	Conductivity (S/m)	Conductivity ranges used in gPC model (S/m)
White matter (WM)	0.126 ( T. A. Wagner et al., 2004 )	0.1 < $\sigma_{WM}$ < 0.4 ( Li et al., 1968 ) ( Nicholson, 1965 ) ( Akhtari et al., 2010 )
Gray matter (GM)	0.275 ( T. A. Wagner et al., 2004 )	0.1 < $\sigma_{GM}$ < 0.6 ( Li et al., 1968 ) ( Ranck, 1963 ) ( Logothetis et al., 2007 ) ( Yedlin et al., 1974 )
Cerebrospinal fluid (CSF)	1.654 ( T. A. Wagner et al., 2004 )	0.8 < $\sigma_{CSF}$ < 1.8 ( Gabriel et al., 2009 ) ( Baumann et al., 1997 )
Spongy bone (SB)	0.025 ( Opitz et al., 2015 )	0.015 < $\sigma_{SB}$ < 0.040 ( Akhtari et al., 2002 )
Compact bone (CB)	0.008 ( Opitz et al., 2015 )	0.003 < $\sigma_{CB}$ < 0.012 ( Akhtari et al., 2002 ) ( Tang et al., 2008 )
Scalp	0.465 ( T. A. Wagner et al., 2004 )	0.2 < $\sigma_{scalp}$ < 1 ( Gabriel et al., 2009 ) ( Yamamoto & Yamamoto, 1976 ) ( BURGER & van MILAAN, 1943 )
Eyeballs	0.5 ( Opitz et al., 2015 )	N/A
Veins & Arteries (“blood”)	0.6 ( Gabriel et al., 2009 )	N/A
Rectus muscles	0.16 ( Gabriel et al., 2009 )	N/A
Electrode rubber	29.0	N/A
Conductive gel	1.0	N/A

### 2.5 Sub-study 1: Relevance of considering anisotropic conductivity in electric field simulations

We aimed to determine how much the inclusion of conductivity anisotropy of brain tissue changes the calculated tES-induced**E**- and*B_z_*-fields compared to simulations with isotropic conductivities. In particular, we were interested to learn how this effect compares to the uncertainty of the**E**- and*B_z_*-fields that arises from our insufficient knowledge of the tissue conductivities. For that, we performed systematic uncertainty analyses to determine the range of feasible**E**- and*B_z_*-fields, given the range of tissue conductivities reported in the literature, using a Generalized Polynomial Chaos (gPC) expansion method (details described next). We ran three separate uncertainty analyses for each head model and electrode montage, (1) simulating all tissues as isotropic, (2) simulating GM and WM as anisotropic using the ‘direct mapping’, and (3) using the ‘volume normalized’ mapping for GM and WM. This allowed us to quantify the uncertainty ranges of the**E**- and*B_z_*-fields, and to determine how much the ranges were affected by the choice of anisotropic brain conductivities. In addition, gPC provides estimations of the average**E**- and*B_z_*-fields that are to be expected for the given range of tissue conductivities.

In order to get insight into the relevance of including brain conductivity anisotropy in the simulations, we determined the differences of the average**E**- and*B_z_*-fields between cases (i) to (iii) and compared them to the uncertainty ranges for each of the cases. The following subsections first describe the gPC method for uncertainty analyses before then providing details of the simulation settings for sub-study 1.

#### 2.5.1 Generalized Polynomial Chaos (gPC) expansion method

Details of the gPC method and its adaptation to tES field simulations are covered in the original articles (G. B. Saturnino, Thielscher, et al., 2019;Weise et al., 2015,^2020^). Briefly, the core concept of gPC is to approximate the functional dependence between the random variables (here: the tissue conductivities assembled in vectorσ) and the quantity of interest (here: the magnitude of the tES-induced**E**-field |**E**|, or the B_z_component of the resulting magnetic field) by a compact analytical model consisting of orthogonal polynomial basis functions:

$$q(\sigma )\approx \sum_{\alpha \in \text{A}}\;u_{\alpha }\Psi_{\alpha }(\sigma )$$ (5)

The quantity of interestq(σ)is a vector of the |**E**| or B_z_values at the relevant positions, that is, at the nodes of the gray matter central surfaces or the voxel positions of the MREIT imaging slice. The functions$\Psi_{\alpha }(\sigma )=\Pi_{\text{i}=1}^{\text{d}}\psi_{\alpha_{\text{i}}}^{\text{i}}(\sigma_{\text{i}})$are the joint polynomial bias functions of the gPC. They are composed of polynomials$\psi_{\alpha_{\text{i}}}^{\text{i}}(\sigma_{\text{i}})$which are separately defined for each of the d tissue conductivities. The multi-index**α**is a vector of length d that states the degrees of the individual polynomials of the joint basis function. The polynomials are multiplied with the position-dependent gPC coefficients$u_{\alpha }$to obtain an estimate of the quantity of interestq(σ). GPC requires that the uncertainties of the random variables are described as mutually independent probability density functions (PDF), whereby the polynomials are chosen to be orthogonal in the normed spaced induced by the PDFs (Askey & Wilson, 1985). Here, we model the tissue conductivities as uniform distributions covering the conductivity ranges reported in literature.

During an initial training procedure, the gPC coefficients are determined along with the construction of the polynomial basis to approximate the quantity of interest. Starting from an initial set of basis functions, the coefficients are fitted by sampling from the probability density distributions of the tissue conductivities and determining the correspondingq(σ)(i.e., the |**E**|- or B_z_-fields) via FEM (Section 2.4). A regression method is used to obtain the coefficients$u_{\alpha }$based on a set of values forσandq(σ), and the error between the gPC approximation and the trueq(σ)is estimated using a k-fold cross-validation scheme. The model order is successively increased until a predefined tolerance level of 0.01 is achieved.

After training, the gPC model can be used for a computationally efficient calculation of the |**E|**- and B_z_-fields for new samples of the tissue conductivities (as long as the fall within the previously specified ranges). In practice, this can replace the costly FEM calculations in cases which require many evaluations of the transfer function for varying input parameters. In addition, as the gPC method employs an analytical model, certain statistical properties of the quantity of interest can be efficiently evaluated directly from the coefficients of the polynomial expansion. Of interest for our aims, this includes the expectation (i.e., mean)**μ**and variance**ν**:

$$\mu =u_{\alpha_{1}}$$ (6)

$$\nu =\sum_{\alpha \in \text{A}\backslash \alpha_{1}}{\left(u_{a}\right)}^{2}$$ (7)

In addition, the uncertainty of the |**E**|- and Bz-fields can be evaluated using Sobol indices. The Sobol indices decompose the total variance of the quantity of interest into components that can be attributed to individual random variables (i.e., specific tissue conductivities), and thus enables conclusions on how much specific tissue compartments contribute to the total variance of the |**E**|- and Bz-fields.

#### 2.5.2 Simulation details

The A-P and R-L montages were modeled with circular rubber electrodes (3.5 cm diameter). For the M1-SO montage, rectangular 5 × 5 cm² electrodes were modeled targeting the M1, and the return electrode (rectangular 5 × 7 cm²) was placed over the supraorbital region. The lower and upper boundaries of the uniform distributions used to model the feasible conductivity ranges are listed inTable 1, together with the corresponding experimental studies from which these values were extracted. We considered only studies which measured fresh or live tissue (preferably human) at low frequencies near body temperature, preferentially using a four-electrode setup.

For each setup, gPC models of the**E**- and*B_z_*-fields were determined separately for considering 1) isotropic and homogeneous conductivities, 2) anisotropic GM and WM conductivities according to the ‘direct mapping’ method, and 3) anisotropic GM and WM conductivities based on the ‘volume normalized’ method. During training of the gPC models for cases 2 and 3, the GM and WM conductivity values drawn from the PDFs were converted into anisotropic GM and WM conductivities by applying the corresponding equations outlined inSection 2.3and the DTI data of the participants.

For visualizations, the expected average**E**-field magnitude$\left|{\text{E}}_{avg}\right|$, its standard deviation (STD), and its Sobol indices were extracted from the gPC models at each location of the middle GM surface. In addition, differences between average**E**-field magnitudes of the gPC models were calculated to determine the impact of brain conductivity anisotropy on the**E**-field distributions.

To enable group comparisons, a mean standard deviation was calculated across the middle GM surface of each participant for each gPC model. This value was expressed as percentage of the mean**E**-field magnitude across the middle GM surface:

$$\text{relative STD}=\frac{\sum_{i=1}^{K}\text{STD}(i)}{\sum_{i=1}^{K}\;\left|E_{avg}(i)\right|}\times 100$$ (8)

where K denotes the number of surface nodes. In addition, the mean of the absolute differences between the**E**-field magnitudes (denoted by |**E**|) of the gPC models with anisotropic versus isotropic brain conductivities were determined across the nodes of the middle GM surface (i indexes the surface nodes), and expressed as percentage of the mean**E**-field magnitude:

$$\text{relative difference}=\sqrt{\frac{\sum_{i=1}^{K}{\left(\left|E_{avg}^{AnISO}(i)\right|\;-\;\left|E_{avg}^{ISO}(i)\right|\right)}^{2}}{\sum_{i=1}^{K}{\left|E_{avg}^{ISO}(i)\right|}^{2}}}\times 100$$ (9)

Relative standard deviations and mean field differences were also computed for the**E**-field magnitude in the GM volume, and the normal component of**E**-field in the middle GM surface. Finally, the average*B_z_*-fields were extracted in a 2D slice approximating the position and orientation of the MREIT measurements used in sub-study 2. Again, relative differences between the average*B_z_*-fields of the different gPC models were calculated to determine the impact of brain conductivity anisotropy on the current-induced magnetic fields (substituting$\left|{\text{E}}_{avg}(i)\right|$by*B_z_*(*i*) inEquation 6, whereby*i*denotes voxel indices in a mask covering GM, WM, and CSF in the 2D slice). Only the results for the ‘direct mapping’ are reported, as both mapping methods had similar impact on the*B_z_*-fields.

### 2.6 Sub-study 2: Impact of anisotropic brain conductivity on the fit to experimental MREIT data

We aimed to determine whether modeling the conductivity of the brain as anisotropic improves the fit of the simulated current-induced magnetic fields to the measured data, compared to the use of isotropic brain conductivities. We used our prior study as a starting point that revealed that the fit of simulated and measured*B_z_*-fields could be improved by optimizing the ohmic conductivities of the volume conductor model, rather than assuming “standard” literature conductivities (Eroğlu et al., 2021). Briefly, this approaches optimizes the tissue-specific conductivities ($\sigma =[\sigma_{scalp},\;\sigma_{WM},\;\sigma_{GM},\;\sigma_{CSF},\;\sigma_{CB},\sigma_{SB}]$) using the sequential least squares programming algorithm with the goal to minimize the relative error between simulated magnetic field$B_{z}$given by the head model and the reference$B_{z}^{ref}$from the human MREIT data:

$$\begin{array}{l} Minimize\;\delta_{B_{z}}=\sqrt{\frac{\sum_{i=1}^{N}{\left(B_{z}(\sigma ,i)-B_{z}^{ref}(i)\right)}^{2}}{\sum_{i=1}^{N}{\left(B_{z}^{ref}(i)\right)}^{2}}}\times 100\\ \;\;\;\;\;\;\;\;\;\;\;\;\;\;\;\;\;\;\;\;\;\;\;\;\;\;\;\;\;\;\;\;\;\;\;subject\;to:\;\sigma_{low}<\sigma <\sigma_{high} \end{array}$$ (10)

As the optimization requires repeated simulations of*B_z_*-field maps for varying conductivities, we used the trained gPC models from sub-study 1 to reduce the number of required FEM simulations of the*B_z_*-fields. The approach implements constraints on the minimal and maximal conductivities to reduce the likelihood of overfitting that could occur, for example in case of systematic differences of the volume conductor models to the true head anatomy. We employed the same conductivity ranges as used for the gPC in sub-study 1 (listed inTable 1), and we imposed$\sigma_{GM}$>$\sigma_{WM}$as an additional constraint.

Using the stray-field-corrected*B_z_*data of the 5 participants (each with A-P and R-L montages) as$B_{z}^{ref}$, we applied the optimization algorithm (Eq. 7) for 1) isotropic and homogeneous model conductivities, and 2) anisotropic GM and WM conductivities according to the ‘direct mapping’ method. For the latter case, the GM and WM conductivity values were converted into anisotropic GM and WM conductivities by applying the corresponding equations outlined inSection 2.3. We were interested to see whether using anisotropic brain conductivities would lead to a smaller remaining relative error$\delta_{B_{z}}$after optimization. To test this, we used a 3-way repeated-measures ANOVA (python 3.9, statsmodels 0.14.1) with the factors “conductivity source” (Literature, Optimized), “conductivity type” (ISO, AnISO), and “montage” (AP, RL). Only the results for the ‘direct mapping’ are reported here, as using the ‘volume normalized’ approach gave similar results.

### 2.7 Sub-study 3: Reconstruction of conductivity using DT-magnetic resonance electrical impedance tomography (DT-MREIT) for human head anatomies

The approach used in sub-study 2 only optimizes the GM and WM conductivity values globally for the complete gray and white matter compartments. Anisotropic brain conductivities are then estimated from those values using the fixed conversion schemes outlined inSection 2.3. Ideally, as these schemes still lack validation, one would rather achieve a voxel-by-voxel reconstruction of the conductivity from the measured MREIT data. For this reason, the DT-MREIT algorithm (Kwon et al., 2014) was introduced, which calculates a voxel-wise (rather than global) scaling of the water diffusion tensors from the MREIT data. The DT-MREIT algorithm has been validated for a simplified phantom geometries. Briefly, the phantom includes two pairs of attached electrodes on its lateral surfaces to inject predominantly horizontal currents, and four additional objects: One isotropic cylinder object and three anisotropic objects with different directional diffusion tensors. The background of the model was set to a constant isotropic conductivity (seeSupplementary Material Bfor further details). Here, we evaluate its performance for human head MREIT data, as a putative alternative to the approach used in sub-study 2. Using the human head model ‘*ernie*’ from the SimNIBS example dataset with known conductivity distributions as ground truth, we test how well these conductivities are reconstructed by DT-MREIT from simulated current flow and*B_z_*data.

#### 2.7.1 DT-MREIT algorithm

The approach uses two successive steps: 1) the reconstruction of the current densities from the measured*B_z_*-fields, followed by 2) the reconstruction of the conductivity tensors from the calculated current densities and the DTI data. In order to reconstruct current densities$J^{rec}$from the measured*B_z_*-fields, the projected current density algorithm was proposed (Jeong et al., 2014):

$$J^{rec}=\;J^{0}+\;\frac{1}{\mu_{0}}\;\left[\frac{\partial (B_{z}-B_{z}^{0})}{\partial y},\;\frac{\partial (B_{z}-B_{z}^{0})}{\partial x},\;0\right]$$ (11)

where$\mu_{0}$is the magnetic permeability of free space,$J^{0}=[J_{x}^{0},J_{y}^{0},J_{z}^{0}]$is the simulated current densities for a uniform conductivity assigned to the complete volume conductor model, and$B_{z}^{0}$is the z-component of the respective magnetic flux density. The algorithm is based on the assumption that$\frac{\partial B_{x}}{\partial z}\;\approx \;\frac{\partial B_{x}^{0}}{\partial z}\;$and$\frac{\partial B_{y}}{\partial z}\;\approx \;\frac{\partial B_{y}^{0}}{\partial z}$(Eroğlu et al., 2021;Jeong et al., 2014).

The DT-MREIT algorithm is based on the assumption that the water diffusion tensorDmeasured by DTI and the ohmic conductivity tensorσare linearly related

σ=ηD (12)

wherebyηis a position-dependent scaling factor or “diffusivity ratio” map. The goal of the DT-MREIT algorithm is to recoverηfrom the reconstructed current density maps of at least two independent current injections (i.e., differing in their electrode positions).

Using Ohm’s lawJ=σE=ηDEand the fact that**E**is curl-free in the electrostatic case, the following relation can be derived

∇×(D−1 J)=∇×ηE=∇η×E=∇ηη ×(η E)=   ∇logη ×​ (D−1 J)  (13)

whereby∇×is the curl operator. Focusing on an axial (i.e., xy) plane and usingEquation 10, the two transversal parts of the gradient vector∇logηcan be recovered from two linearly independent current injections ($J^{rec,1}$and$J^{rec,2}$) by solving the following matrix system (Kwon et al., 2014):

$$\left(\begin{array}{c} {\left({\text{D}}^{-1}\;{\text{J}}^{rec,1}\right)}_{y}-{\left({\text{D}}^{-1}\;{\text{J}}^{rec,1}\right)}_{x}\\ {\left({\text{D}}^{-1}\;{\text{J}}^{rec,2}\right)}_{y}-{\left({\text{D}}^{-1}\;{\text{J}}^{rec,2}\right)}_{x} \end{array}\right)\left(\begin{array}{c} \frac{\partial \log\eta }{\partial x}\\ \frac{\partial \log\eta }{\partial y} \end{array}\right)\approx \;\left(\begin{array}{c} \frac{\partial \left({\text{D}}^{-1}\;{\text{J}}^{rec,1}\right)}{\partial x}-\frac{\partial \left({\text{D}}^{-1}\;{\text{J}}^{rec,1}\right)}{\partial y}\\ \frac{\partial \left({\text{D}}^{-1}\;{\text{J}}^{rec,2}\right)}{\partial x}-\frac{\partial \left({\text{D}}^{-1}\;{\text{J}}^{rec,2}\right)}{\partial y} \end{array}\right)\;$$ (14)

Finally,η is reconstructed from the recovered parts of∇logηas the last step (Kwon et al., 2014) and applied toEquation 9to get the ohmic conductivity tensors.

#### 2.7.2 Human head model

The publicly available ‘*ernie*’ model (simnibs.github.io/simnibs/build/html/dataset.html) was used. Diffusion tensors were prepared from the raw diffusion MRI with a modified version of the SimNIBS script*dwi2cond*so that the CSF and intracranial blood vessels were maintained (Fig. 6B). Conductivity tensors for GM and WM were then created from the diffusion tensors by linearly scaling the diffusion tensors (separately for each of the two regions) so that their average conductivities matched those derived from the literature (Fig. 6C). Specifically, the region-average of the geometric means of the eigenvalues of the conductivity tensors was matched to the conductivities derived from the literature (Table 1). CSF was modeled as isotropic and homogenous tensors using conductivities derived from the literature. This procedure ensured that the relative conductivity ratios between GM, WM, and CSF corresponded to the ratio between the conductivities derived from the literature for these tissues, while allowing for anisotropic and inhomogeneous conductivities within GM and WM. The resulting conductivity tensor image (CTI) was slightly smoothed with a 1 mm Gaussian filter to avoid step-wise conductivity changes (and by that also discontinuities in the simulated current densities), as pilot testing showed that this markedly improved the performance of the DT-MREIT algorithm. This slightly altered the average tissue conductivities within each compartment, compared to the literature values (2nd column inTable 2). The conductivities of the remaining tissue regions were modeled as isotropic and homogenous using their conductivities derived from the literature. In the subsequent tests, the CTI data were used as ground truth, and it was assessed how well the DT-MREIT algorithm could recover it from the diffusion tensors and the supplied current densities.

**Table 2.** Geometric means of the eigenvalues of the conductivity tensors in GM, WM, and CSF of the head model (in [S/m]).

Tissue	Ground truth	AnISO	ISO	*Jproj*
WM	0.17 ± 0.10	0.17 ± 0.10	0.22 ± 0.09	0.35 ± 0.09
GM	0.44 ± 0.22	0.46 ± 0.20	0.47 ± 0.17	0.48 ± 0.12
CSF	1.42 ± 0.36	1.10 ± 0.64	1.05 ± 0.58	0.86 ± 0.51

The ground truth values (column 2) correspond to the conductivities used for the simulations. Columns 3 to 5 list the reconstructed values determined by the DT-MREIT algorithm for different current density information used as input (“AnISO”: current density for anisotropic ground-truth brain conductivities. “ISO”: current densities for matched isotropic brain conductivities. “*Jproj*”: current densities determined via the projected current density algorithm from the ground-truth*B_z_*data). Listed are the averages ± standard deviation within each tissue (units: S/m).

For comparisons, the conductivities of GM, WM, and CSF were modeled as isotropic and homogenous tensors, using their conductivities derived from the literature, and the resulting CTI image was slightly smoothed with a 1 mm Gaussian filter.

#### 2.7.3 Simulation details

The evaluation was performed for two montages (AP and RL) and current strengths of 1 mA baseline-to-peak in both cases. We used the DT-MREIT algorithm implemented in the CoReHA toolbox (Sajib et al., 2017), based on Matlab (The MathWorks, MA, USA), to reconstruct the conductivity distribution in the brain and CSF within a central axial slice (Fig. 6A). We evaluated the performance of the algorithm in three cases (Fig. S9gives a graphical summary of the workflow):

1. “*AnISO*”, in which the current densities that were calculated for anisotropic brain conductivities (using the ground truth CTI data) were directly used as input to the DT-MREIT algorithm.
2. “*ISO*”, which used the current densities calculated for matched isotropic brain conductivities as input to the DT-MREIT algorithm. By that, we aimed to assess whether the algorithm is sensitive to the differences in the current densities caused by anisotropic versus isotropic and homogeneous brain conductivities.
3. “*Jproj*”, in which the*B_z_*-fields of the simulated current densities for anisotropic brain conductivities were calculated and used as input to the projected current density algorithm. The DT-MREIT algorithm was then applied to the results of the former step. While case 1 represents a best-case scenario where the current density is exactly known, case 3 mimics the realistic scenario where the current density has to be recovered from the measured*B_z_*data.

The performance of the algorithm was evaluated based on the relative error of the geometric means of the eigenvalues of the conductivity tensors determined by DT-MREIT, compared to their ground-truth values. As the algorithm applies a common scaling to all components of the diffusion tensors, making a component-wise error analysis unnecessary, the geometric means were used to simplify the analyses and visualizations. In addition, the relative error of the estimated scale factorsηcompared to their ground truth values was calculated.

The DT-MREIT algorithm requires selection of two free parameters, a regularization parameter (parameter ξ of equation 38 inKwon et al., 2014) and a scaling factor between the diffusion and conductivity tensors at the region boundary (log η_ext_in equation 32 inKwon et al., 2014). As their optimal values were unknown, the parameters were systematically optimized for all cases separately (using fminsearch of Matlab) to minimize the relative error of the geometric means of the eigenvalues of the conductivity tensors. That is, all results correspond to the ideal situation that the best scaling factor and regularization parameter are known, and that the provided current densities are noise-free.

The good performance of the DT-MREIT algorithm for a simplified phantom geometry, similar to that used inKwon et al. (2014), was confirmed in pilot tests (Supplementary Material B).

## 3 Results

### 3.1 Relevance of considering anisotropic conductivity in electric field simulations (sub-study 1)

**Fig. 2.**
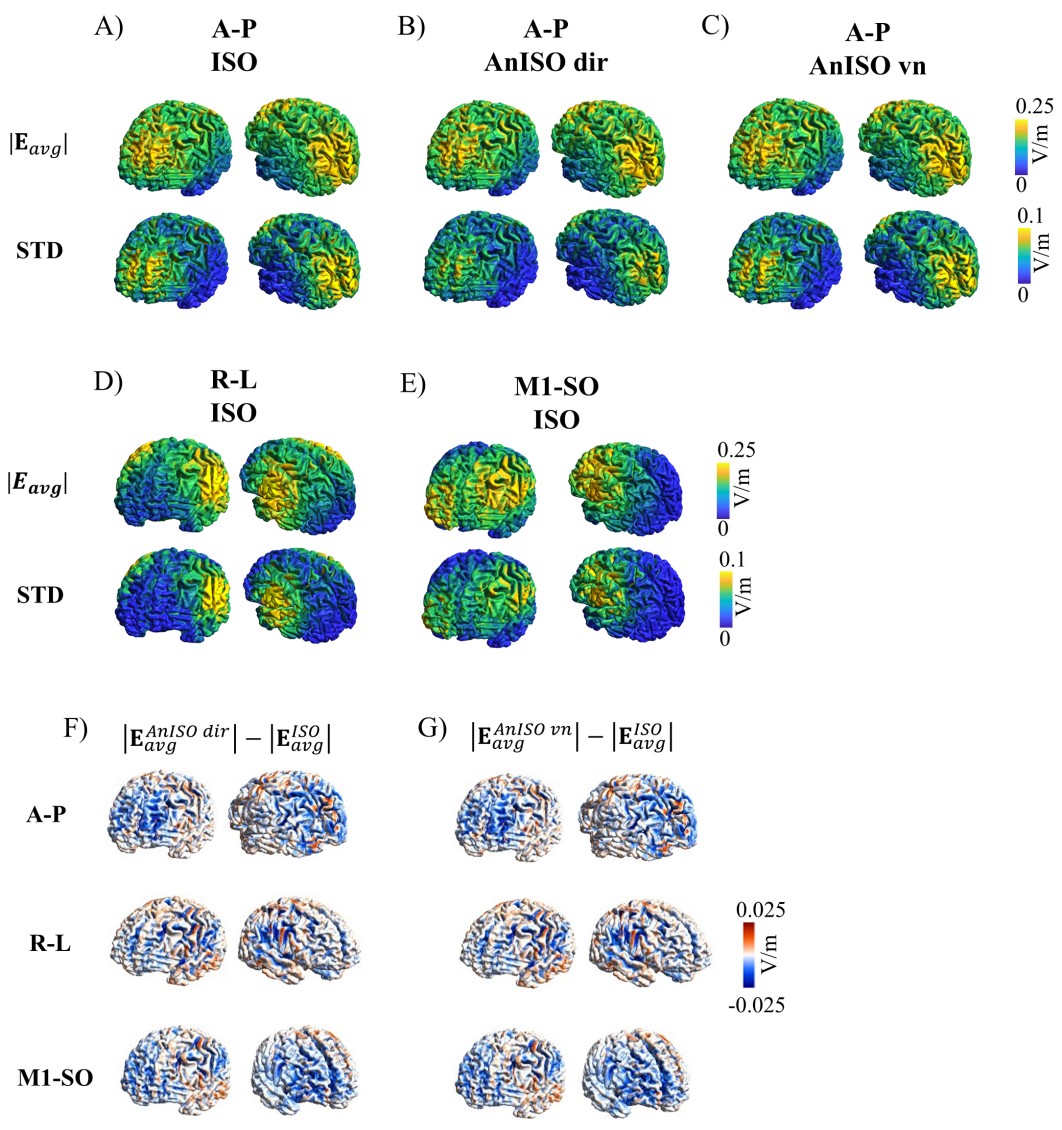
Simulated electric field magnitude on the middle gray matter surface of a representative participant. The gPC method was used to evaluate the electric field as a function of conductivities in three different montages and isotropic and anisotropic brain conductivities: (A) Isotropic conductivities (ISO; A-P montage), (B) anisotropic brain conductivities based on the ‘direct mapping’ method (AnISO dir;; A-P montage), and (C) anisotropic brain conductivities according to the ‘volume normalized’ method (AnISO vn; A-P montage). (D) Isotropic conductivities (ISO; R-L montage); (E) Isotropic conductivities (ISO; M1-SO montage); The upper and lower rows show the expected average (|${\text{E}}_{\text{avg}}$|) and the standard deviation (STD) of the electric field strength for the uncertainty ranges of the conductivities defined inTable 1. For the Sobol indices that indicate the contribution of the different tissue compartments to the observed uncertainty, seeFigure S1. For the complete results of the R-L and M1-SO montages for the same participant, seeFigures S2 and S3. (E) Differences between |${\text{E}}_{\text{avg}}$| calculated using anisotropic (‘direct mapping’ method) versus isotropic brain conductivities for the three different montages. (F) Differences between results based on anisotropic (‘volume normalized’ method) versus isotropic brain conductivities for the three montages.

We aimed to systematically investigate the impact of anisotropic conductivities on the simulated tES-induced electric fields in brain gray matter. The gPC results for the electric fields induced by the A-P montage at the middle GM matter surface of a representative participant are shown inFigure 2Afor isotropic conductivities, inFigure 2Bfor anisotropic conductivities according to the ‘direct mapping’ method, and inFigure 2Cbased on the ‘volume normalized’ method. The first row shows the average**E**-field strength that is to be expected given the PDFs defined for the tissue conductivities. The second row depicts the respective standard deviation (STD). The results reveal a strong similarity of the average distributions of the electric field of the isotropic model to the anisotropic ones. In contrast, the calculated electric fields suffer from a large uncertainty due to the uncertainty of the tissue conductivities, as shown by the STD results. These observations were consistent for each of the three simulation set-ups and all participants (seeFigs. S2 and S3for the results of the R-L and M1-SO montages;Fig. S4shows the results for the A-P montage for the remaining participants). Additional analyses of the Sobol indices identify the conductivity uncertainties of GM, compact bone, and scalp as the main contributors to the uncertainty of the**E**-field (Figs. S1–S3).Figure 2D and Eshow the differences between the average**E**-field strengths obtained for simulations using the ‘direct mapping’ and ‘volume normalized’ methods, respectively, and results obtained for isotropic brain conductivities for all three montages. They visually confirm that the differences are small compared to the STD results.

**Fig. 3.**
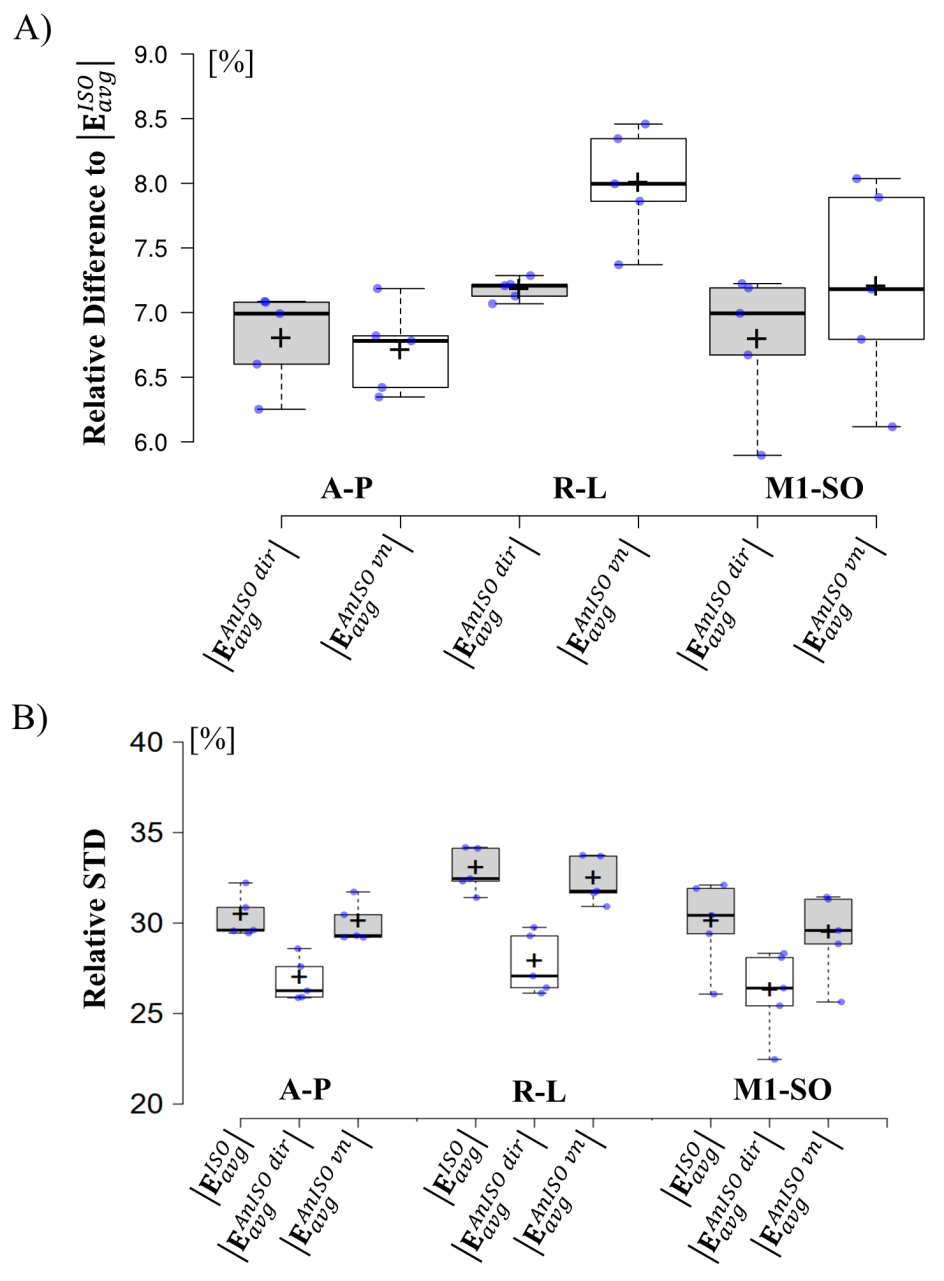
Group results for the simulated electric fields with isotropic and anisotropic conductivities. (A) Relative differences between average electric field magnitudes calculated for anisotropic versus isotropic conductivities for the 5 participants. (B) Relative standard deviations, estimated via the gPC method, of the electric field magnitudes for the 5 participants. Box plots: Whiskers extend to data points that are the 5th and 95th percentile, together with the mean (+) and median (-) across participants. Individual data points are shown as blue dots.

We aimed to quantitatively relate the differences of the E-field strengths at the central GM surfaces for anisotropic versus isotropic brain conductivities (usingEq. 6to evaluate relative differences,Fig. 3A) to the uncertainty of the electric fields caused by uncertain ohmic conductivities (usingEq. 5to evaluate relative STDs,Fig. 3B) on the group level across the 5 participants. For all three montages, the median of the relative STD across the five participants robustly exceeds 25% (Fig. 3B). On the other hand, the median relative differences stay below 8% (Fig. 3A; median: A-P montage: |${\text{E}}_{avg}^{AnIso\;dir}$|: 7.0% and |${\text{E}}_{avg}^{AnIso\;vn}$|: 6.8%, for R-L: |${\text{E}}_{avg}^{AnIso\;dir}$|: 7.2% and |${\text{E}}_{avg}^{AnIso\;vn}$|: 8.0%, for M1-SO: |${\text{E}}_{avg}^{AnIso\;dir}$|: 7.0% and |${\text{E}}_{avg}^{AnIso\;dir}$|: 7.2%). An identical analysis was performed for the**E**-field magnitude$\left(E_{magn}\right)$in the GM volume and normal component of the**E**-field$\left(E_{norm}\right)$at the middle GM surface (Fig. S5). The relative differences for$E_{magn}$in the GM volume were in the same r mathvariant="bold"ange as those found at the cetnral GM surface, and slightly larger for$E_{norm}$(median relative differences between 7% and 9.5% in the former, and between 10% and 11% in the latter). Similarly to the$E_{magn}$at the middle GM surfaces, the relative STDs of$E_{magn}$in the GM volume robustly exceeded 25% on average across the 5 participants. For$E_{norm}$at the central GM surfaces, the relative STD on average exceeded 30%. To support the results obtained for the 5 enrolled participants, relative STDs and differences for the average$E_{magn}$in the middle GM surface were computed for 20 randomly selected HCP subjects (Fig. S6). The results were in the same range as those obtained for the 5 recruited subjects, with only slightly larger relative differences for the average$E_{magn}$(median values below 9%). Collectively, the analyses reveal a small to moderate impact of brain conductivity anisotropy on the electric field distributions in gray matter that is far exceeded by the uncertainty of the electric field due to not well-known tissue conductivities.

In general, the relative STD for GM are smaller for anisotropic conductivities derived by the ‘direct mapping’ method than for the other two cases.

SeeFigure S5for a discussion of the cause and additional control analyses to confirm the validity of the results. Importantly, also for the ‘direct mapping’ method, the STD of the electric field in GM clearly remains higher than the electric field differences between the isotropic and anisotropic models.

**Fig. 4.**
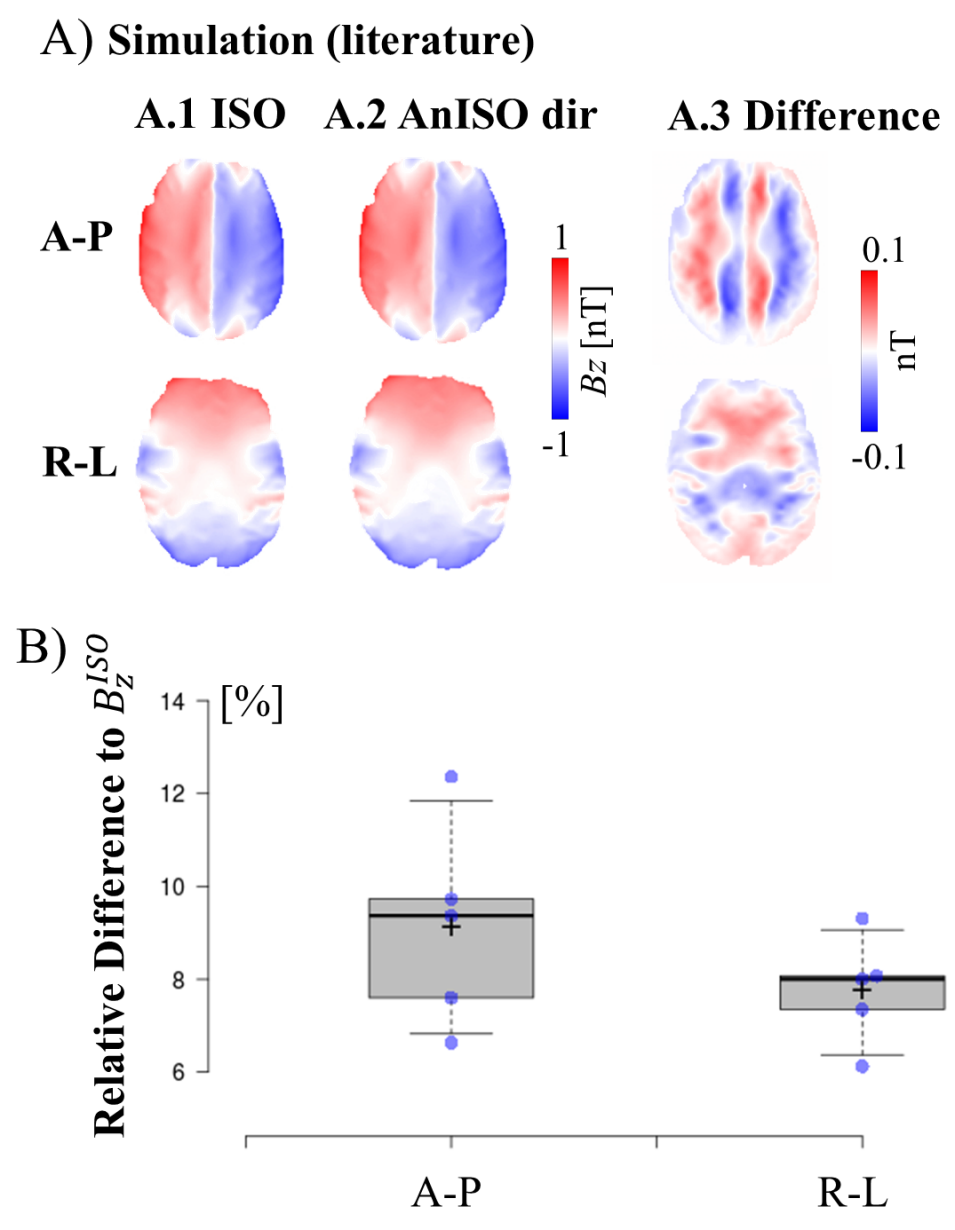
Differences between the simulated B_z_-fields with isotropic and anisotropic conductivities. (A) B_z_distributions for horizontal slices covering the bottom part of the brain of a representative participant for the A-P and R-L montages. A mask was applied to extract GM, WM, and CSF. The results for isotropic (A.1) and anisotropic (‘direct mapping’ method) (A.2) literature-based conductivities (Table 1), and the difference between the simulated*B_z_*-fields with isotropic and anisotropic conductivities (A.3) are shown. (B) Relative differences between the B_z_-fields calculated for anisotropic (AnISO dir) versus isotropic conductivities for the 5 participants. Box plots: Whiskers extend to data points that are the 5th and 95th percentile, together with the mean (+) and median (-) across participants. Individual data points are shown as blue dots. For the individual results of all participants, seeFigure S6.

Figure 4shows the differences between$B_{z}$-fields in horizontal brain slices that were simulated using head models with isotropic brain conductivities versus anisotropic brain conductivities according to the ‘direct mapping’ method for the 5 participants.Figure 4Ashows the results of a representative participant. The group results inFigure 4Bindicate a median relative difference of 9.2% (6.6–12.4%) for the A-P montage and 7.8% (6.1–9.3%) for the R-L montage. In order to confirm that these results are representative for the differences in the overall brain volume, we re-evaluated the participant with the largest differences again using a 3D brain mask. The results (3D: A-P 7.7%, R-L 6.7%) suggest that the differences in the 2D slices that were aligned to the electrode positions tend to be higher than on average in the whole brain. A similar analysis was performed for the 20 HCP subjects, where the$B_{z}$-fields were simulated in horizontal slices going through both electrodes (R-L) or centered in between the electrodes (A-P). The results indicate a median relative difference of 8.9% (5.8–16.9%) for the A-P montage and 11.6% (7.9–16.2%) for the R-L montage (seeSupplementary Material A;Fig. S9). In summary, the analyses reveal that the impact of brain conductivity anisotropy on the simulated$B_{z}$-field distributions is in a similar range as its impact on the simulated electric fields.

### 3.2 Relevance of considering anisotropic conductivity in reconstructing experimental MREIT data (sub-study 2)

**Fig. 5.**
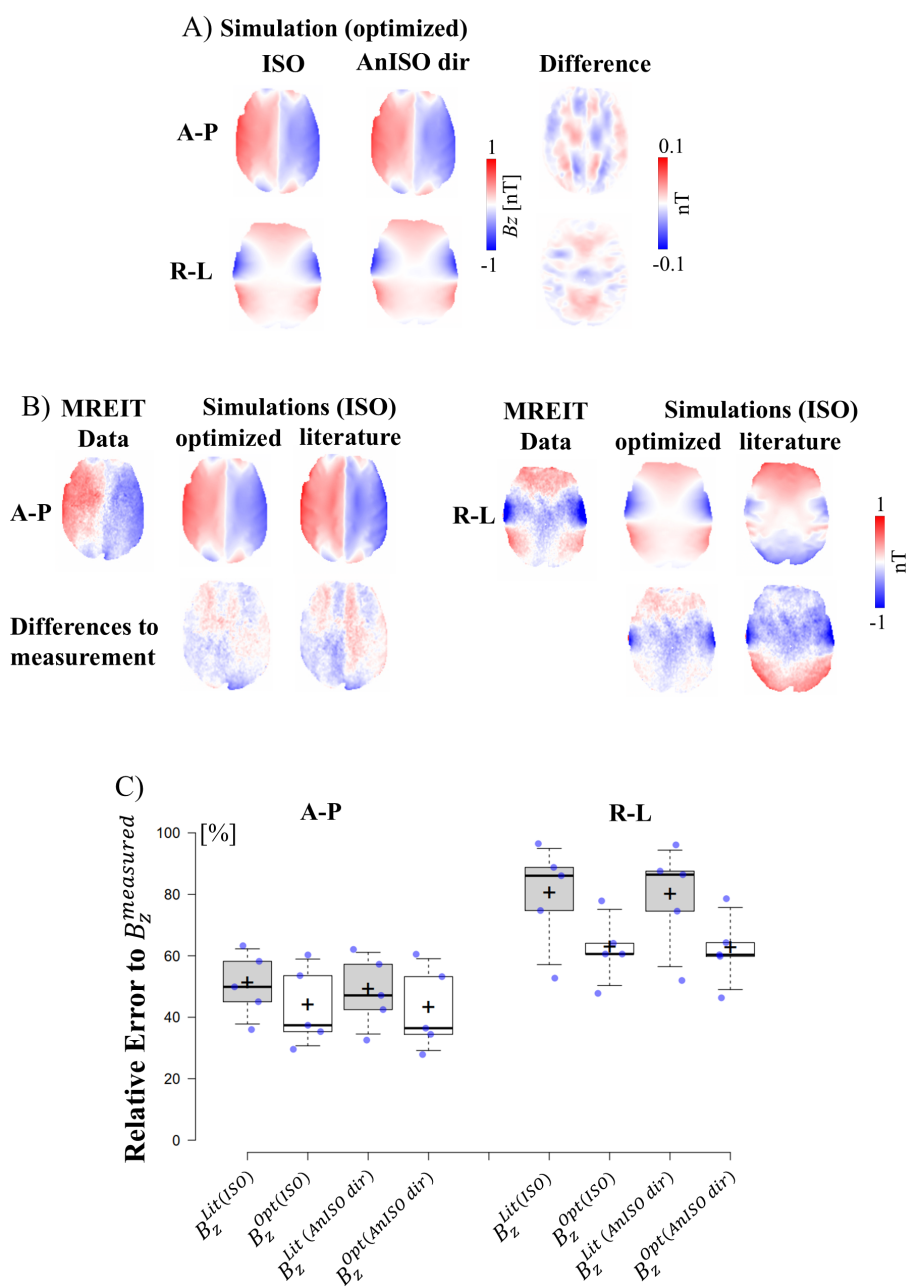
Errors between the simulated and measured B_z_-fields. (A) Simulated B_z_distributions for the same horizontal slices and representative participant as inFigure 4A, but for optimized conductivities. (B) Measured B_z_distributions of the participant and differences of the simulated fields to the measured data for optimized and literature-based conductivities. The differences are shown only for isotropic conductivities here, as those for the anisotropic conductivities are very similar. (C) Relative errors of the simulated versus measured*B_z_*distributions (Eq. 7) for the 5 participants. The errors are reported for four conductivity settings: 1) Lit(ISO): Standard isotropic conductivities (Table 1); 2) Opt(ISO): Optimized isotropic conductivities, maximizing the match between simulated and measured B_z_-fields; 3) Lit(AnISO dir): Anisotropic conductivities (‘direct mapping’), normalized to match literature conductivities; 4) Opt(AnISO dir): Anisotropic conductivities (‘direct mapping’), optimized to maximize the match between simulated and measured B_z_-fields. Note that the relative error levels are very similar for ISO and*AnISO dir*, indicating that modeling brain conductivity as anisotropic does not improve the fit to the measured B_z_-fields. Box plots: Whiskers extend to data points that are the 5th and 95th percentile, together with the mean (+) and median (-) across participants. Individual data points are shown as blue dots. For the complete individual measured B_z_field distributions, seeFigure S7. The fitted conductivities are summarized inFigure S8.

We aimed to determine whether modeling the conductivity of the brain as anisotropic improves the fit of the simulated current-induced magnetic fields to the measured data, compared to the use of isotropic brain conductivities. To investigate that, we tested whether including anisotropic conductivity results in a lower mismatch between the simulated and measured current-induced*B_z_*-fields.Figure 5Ashows the simulated$B_{z}$-fields for isotropic and anisotropic conductivities (according to the ‘direct mapping’) for the representative participant ofFigure 4A, now after optimization of the tissue conductivities usingEquation 7.Figure 5Bshows the corresponding measured$B_{z}$-fields, and the remaining differences of the simulated to the measured fields (shown for isotropic conductivities only, as the anisotropic conductivities exhibit very similar results). As apparent from the small differences inFigure 5A, the simulated$B_{z}$-fields remain very similar for the isotropic and anisotropic models also after optimization. Conductivity optimization benefits mostly the simulation of the R-L montage, which shows smaller differences to the measurements, compared to simulations using conductivities derived from the literature.

Figure 5Cshows relative errors (evaluated usingEq. 7) for all participants. When using conductivities from the literature, the results show very similar relative error levels for ISO and*AnISO dir*(two gray bar plots for each montage inFig. 5C), with median differences of 1.1% between both (minimum -0.3%, maximum 2.8%). Generally, the relative errors are higher for the R-L montage, in line with our earlier findings (Göksu et al., 2021). Optimizing the tissue conductivities usingEquation 7consistently decreases the error between the simulated and measured results (two white bar plots for each montage inFig. 5C) in comparison with models employing standard conductivities derived from the literature (median 8.1%, minimum 1.6% and maximum 36.0% of differences between Lit and Opt), with simulations of the R-L montages improving on average more. However, the amount of improvement is very similar for models with isotropic and anisotropic brain conductivities, that is, employing anisotropic conductivities had only a minor impact on the reduction of the respective errors. After optimization, the relative error levels for ISO and*AnISO dir*were again very similar, with median differences of 0.3% between both (minimum -0.8%, maximum 0.9%). In line with these observations, the 3-way repeated-measures ANOVA revealed significant main effects for the factors “conductivity source” (Literature vs. Optimized; t(32) = 5.34, p = 7.45e-06), “montage” (AP vs. RL; t(32) = 11.32, p = 9.97e-13), but not for “conductivity type” (ISO vs. AnISO; t(32) = 0.31, p = 0.761). The results of the ANOVA confirm that modeling brain conductivity as isotropic or anisotropic has no significant effect on how well the simulations fit to the measured MRCDI data, while optimizing the conductivities of the tissue compartments consistently improves the fit compared to the use of standard literature conductivities.

### 3.3 DT-MREIT for human head anatomies (sub-study 3)

We evaluated the accuracy of the DT-MREIT algorithm in three cases: 1)*AnISO,*where the algorithm was informed by current densities calculated for the ground-truth conductivity tensors in the brain and CSF; 2)*ISO*, using current densities from simulations with matched isotropic and homogenous brain and CSF conductivities; and 3)*Jproj*, which employed reconstructed current densities ($J_{x}$and$J_{y}$components) using the projected current density algorithm, based on*B_z_*-fields simulated for anisotropic brain conductivities.

**Fig. 6.**
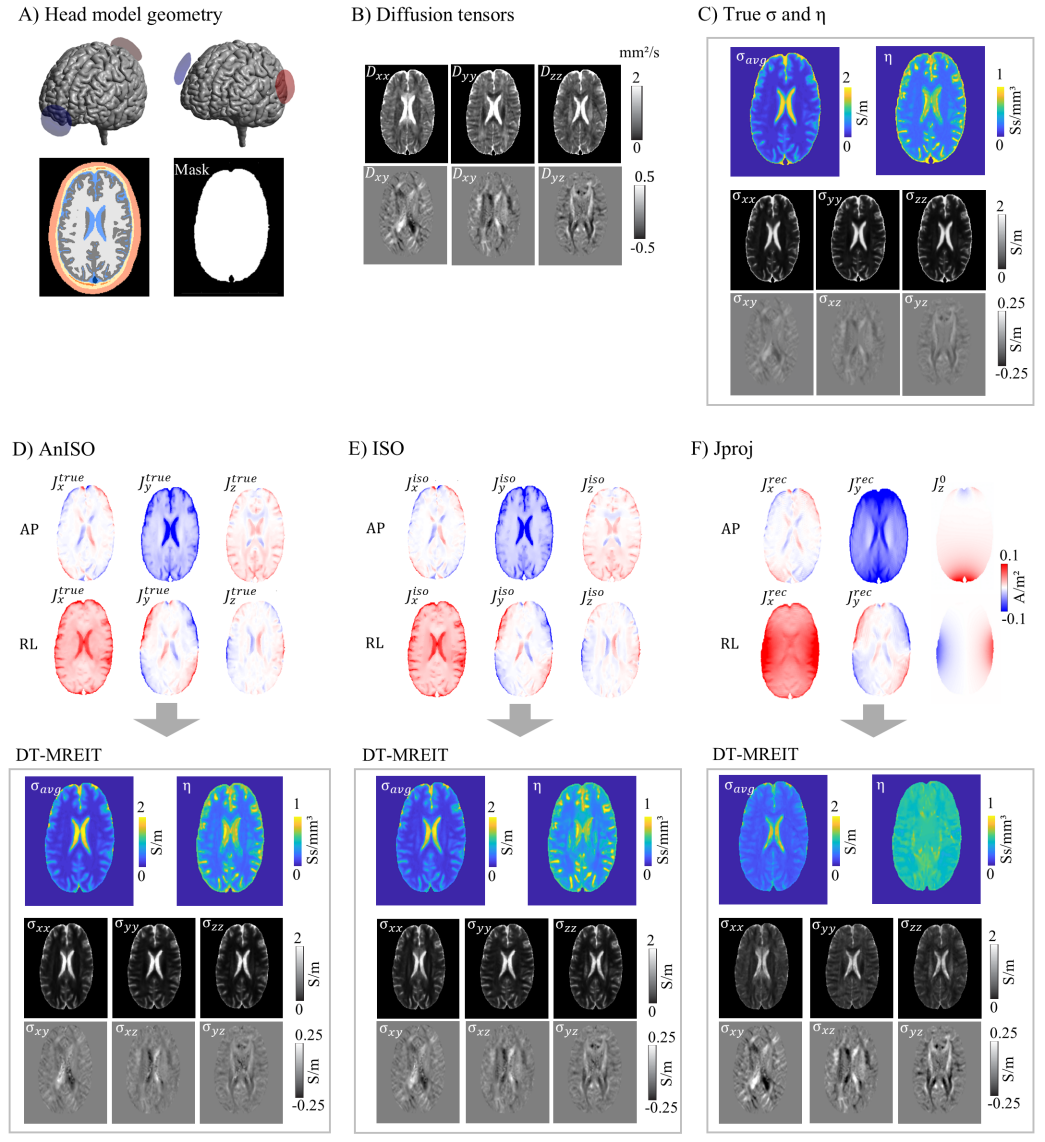
DT-MREIT results (Human head model). (A) The head model ‘ernie’ from the SimNIBS example dataset was used to simulate the**J**- and*B_z_*-fields for two montages (A-P and R-L; 1 mA baseline-to-peak). Top: electrode positions; Bottom: Tissue compartments, and brain and CSF mask. (B) Components of the diffusion tensors**D**in a horizontal slice, used as input to the DT-MREIT algorithm. (C) Ground-truth conductivities σ and scale factors η (σ_avg_denotes the geometric mean of the eigenvalues of the conductivity tensors, σ_xx_, σ_yy_, etc. indicate the tensor components). (D) “AnISO”: Simulated current density distributions for anisotropic (ground-truth) conductivities (top rows) and reconstructed conductivities and scale factors (bottom rows). (E) “ISO”: Simulated current density distributions for matched isotropic conductivities (top rows) and reconstructed conductivities and scale factors (bottom rows). (F) “*Jproj*”: Current densities (J_x_and J_y_components) determined with the projected current density algorithm (Eq. 8) and${\text{J}}_{\text{z}}^{0}$of the head model with uniform conductivity (top rows) and reconstructed conductivities and scale factors (bottom rows).

The current densities for anisotropic (ground-truth) and isotropic brain conductivities are quite similar (relative errors of|J|: AP 11.7% and RL 13.7%; upper halves ofFigs. 6D, E). In contrast, the projected current density algorithm performs less well (Fig. 6F; relative errors for the reconstructed*J_x_*and*J_y_*components: AP 74.9% and RL 81.0%). In addition, it does not recover the*J_z_*component, which has a non-negligible strength in case of the human head model.

DT-MREIT achieves a reasonable accuracy in reconstructing the conductivity tensors and scale factors when supplied with the current densities calculated using the ground-truth conductivity tensors in the brain and CSF (lower half inFig. 6Dfor AnISO;Table 2lists the region-wise averages of the reconstructed conductivities). The relative errors for σ_avg_and η were 41.8% and 34.2%, respectively. Visual inspection suggests that the error is mainly due to a too smooth reconstruction of the conductivity and scale factor images compared to the true images (Fig. 6C), but that the systematic variations of the scale factors between the tissue types are generally well resolved. The accuracy decreases moderately when informing the algorithm using current densities obtained for isotropic and homogenous brain and CSF conductivities (bottom half inFig. 6E). For ISO, the relative errors for σ_avg_and η are 43.6% and 40.1%, respectively (corresponding to increases by 1.8% and 5.9% compared to AnISO). These changes are in the range of the differences between the current densities for anisotropic versus isotropic brain conductivities. Using current densities reconstructed from simulated Bz-fields by means of the projected current density algorithm results in the least accurate DT-MREIT results (bottom half inFig. 6F). For*Jproj*, the relative errors for σ_avg_and η are 51.5% and 62.4% (corresponding to increases by 9.7% and 28.2% compared to AnISO). In particular, the algorithm requires a strong regularization to perform best in this case, resulting in a “flat” scale factor image lacking anatomical detail. That is, due to the strong regularization, the (inaccurate) current densities only weakly influence the solution, resulting in similar scale factors for all voxels.

Overall, while the DT-MREI algorithm demonstrated good reconstruction performance with noise-free and complete input data (ground truth: AnISO), its accuracy significantly declined in a more realistic scenario (*Jproj*). This underscores that the DT-MREIT algorithm requires accurate input data of the tES-induced current densities. However, in practice, the*Jproj*algorithm that is used to estimate the current densities from the raw MREIT data does not work well. This is because the*Jproj*algorithm assumes the z-component of the current flow to be negligible, which is not fulfilled in case of the tES current flow induced in the human head (see our former study for details,Eroğlu et al., 2021).

## 4 Discussion

This study aimed to assess the importance of incorporating brain conductivity anisotropy in tES electric field simulations and the potential for accurately estimating the conductivity anisotropy from MR data. First, we examined the impact of anisotropic conductivities on the simulated tES-induced electric fields and the resulting current-induced magnetic fields. Specifically, we related the effects of anisotropic conductivities to the amount of uncertainty of the electric fields caused by the general uncertainty of the tissue conductivities. We then tested whether including estimates of anisotropic conductivities in the calculations improves the fit between simulated and measured current-induced magnetic fields. Finally, we assessed how much the results of the DT-MREIT algorithm change depending on whether current densities simulated for anisotropic versus isotropic conductivities of the brain are used as input. Overall, the conductivity anisotropy of brain tissue has a small to moderate impact on the calculated tES-induced electric fields in the cortex and the resulting current-induced magnetic fields in the intracranial cavity. There is no clear indication in these data that applying anisotropic conductivities in the simulations would improve the fit between simulated and measured current-induced magnetic fields. The differences between the current flow for anisotropic versus isotropic conductivities of the brain are also too small to markedly affect the results of the DT-MREIT algorithm. In what follows, we discuss the main findings in more detail.

### 4.1 Relevance of incorporating anisotropic brain conductivity in volume conductor models

Our results confirm earlier studies demonstrating that the anisotropic conductivity of brain tissue has only a moderate impact on the electric field in gray matter (Metwally et al., 2015;Shahid et al., 2014;S. Wagner et al., 2014). The conductivity is anisotropic in white matter, but largely isotropic in gray matter. Correspondingly, the electric field in white matter is affected more, though still only moderately (S. Wagner et al., 2014). Employing a systematic uncertainty analysis, our results further show that the electric field variations due to anisotropic brain conductivities are small compared to the general uncertainty of the electric fields that is caused by an insufficient knowledge of the ohmic conductivities of biological tissue. Importantly, the conductivities of several tissue compartments, including the scalp and skull, contribute to the uncertainty of the electric field in gray matter. As such, the accuracy of electric field simulations would benefit more from a better knowledge of the average conductivities of the head tissues than from modeling the brain conductivity as anisotropic. However, the literature about the ohmic conductivities of biological tissues at low frequency is generally sparse, and the available data have been obtained over a large time span using different methodologies and from various species. The conductivity ranges modeled in our uncertainty analysis were based on the few available studies (listed inTable 1), which used fresh or living tissue near body temperature, preferably from humans and measured with a four-electrode setup.

To our knowledge, only two prior studies used measurement data to assess the relevance of modeling anisotropic brain conductivities (Huang et al., 2017;Katoch et al., 2023). Consistent with our comparisons based on current-induced magnetic fields measured by MRI, the choice of anisotropic versus isotropic brain conductivities did not change the model fit to electric fields that were invasively recorded in a patient population (Huang et al., 2017). On the other hand, optimization of the conductivities consistently reduced the differences between simulations and measurements in the study ofHuang et al. (2017), which confirms the importance of an accurate knowledge of the conductivity of all major tissue compartments. This observation is also in line with recent work that aims to estimate individual skull conductivities based on combined EEG and MEG data to reduce the errors of tES simulations (Antonakakis et al., 2020).

In contrast to the above-mentioned literature, another study reports larger differences between simulated tDCS fields induced in the brain for fully isotropic head models compared to models with experimentally determined anisotropic brain and CSF conductivities (Katoch et al., 2023). The anisotropic conductivities were estimated from magnetic resonance electrical properties tomography (MREPT) and diffusion MRI using a method termed conductivity tensor imaging (Sajib et al., 2018). The apparent discrepancy to the findings of the other studies, including ours, was likely caused by the use of conductivity values derived from the literature also for GM, WM, and CSF for the fully isotropic head models inKatoch et al. (2023), rather than employing adapted isotropic conductivities that were matched to the averages of the reconstructed conductivity tensors. That is, while the results ofKatoch et al. (2023)support the relevance of personalized choices of tissue conductivities, they do not prove the importance of employing anisotropic conductivities. In addition, it is relevant to note that the estimated conductivity tensors were also influenced by measurement imperfections and limitations of the employed reconstruction method. In particular, the strongest differences in the simulated current densities (figure 4B inKatoch et al., 2023) occurred at the edge of the brain, where the head models with reconstructed brain and CSF conductivities exhibited higher current densities. Considering that the spatial resolution of the acquired MREPT and diffusion MR images (2 x 2 x 4 mm³) was too low to accurately resolve the gray matter anatomy, we suggest that these results were caused by an overestimation of the conductivities in gray matter due to a blurring of the CSF conductivity tensors into the neighboring GM.

In practice, it remains challenging to ensure that the differences in the fitted or reconstructed conductivities represent true inter-individual differences rather than stemming from noise and biases in the measurements (Eroğlu et al., 2021). Alternatively, estimating group-average conductivities across several participants and montages might help to avoid overfitting, while still improving the overall simulation accuracy compared to “standard” conductivities (Gregersen, 2024;Jiang et al., 2020). Regardless of these methodological aspects, the main point holds that there is little evidence that modeling anisotropic brain conductivity markedly improves the fit to the available invasive and non-invasive recordings.

However, a complementary question that remains to be addressed is how much differences of the**E**-fields of around 8% (as observed between simulations based on isotropic vs. anisotropic brain conductivities) would change the physiological effects of tES. Up to now, only few studies have tested an association between physiological and tES induced**E**-field simulations. In two studies, comparisons of personalized**E**-field simulations with the tDCS effects on motor evoked potential (MEP) amplitudes measured by TMS suggest that**E**-field changes of 8% would alter the tDCS effects on MEP amplitudes by ~5% (Laakso et al., 2019) and ~9% (Mosayebi-Samani et al., 2021). In two another studies, significant correlations of r = -0.66 (R^2^= 0.46) (Nandi et al., 2022) and r = 0.53 (Antonenko et al., 2019) have been reported between interindividual variations in the simulated**E**-field magnitude in the hand area of the motor cortex and the tDCS effects on GABA measured by magnetic resonance spectroscopy. Concluding from the slopes of the reported regression lines, this implies that an 8% change of the**E**-field would correspond to a ~9–10% change of the tDCS effects on GABA. It should be noted that also non-linear relationships between tES intensity and its neurophysiological or behavioral effects have been reported, so that it still needs to be determined how well the above linear dependencies between the**E**-field strength and neurophysiological effects generalize (Boggio et al., 2006;Mosayebi Samani et al., 2019;Nitsche & Paulus, 2000).

### 4.2 Limitations of modeling anisotropic brain conductivity from DTI data

The resolution of standard DTI data as used here is in the order of a few millimeters, which causes partial volume effects between gray matter and the bordering white matter and CSF (seeFig. S10for an example). It can be, therefore, expected that the conductivity transition between gray matter and the more anisotropic white matter is less sharp when estimated from DTI data than the tissue boundary observed in histological data and high-resolution structural MR images. In addition, the diffusivity of gray matter close to CSF is overestimated. When applying the direct mapping method (Eq. 1), this can cause an overestimation of the conductivity at those positions. Given these limitations, some studies restrict the estimation of anisotropic conductivities from DTI data to WM only (S. Wagner et al., 2014). Here, we were interested in the maximal effect of DTI-derived conductivities on the tES current flow and therefore applied the mappings to both GM and WM. In addition, we extracted the electric fields at the central surface in GM to minimize the impact of partial volume effects.

The simulation results are influenced by the choice of the conversion scheme between DTI and conductivity anisotropy. The fixed scaling factor used in the direct mapping method introduced byTuch et al. (2001)can result in unlikely conductivity ranges (e.g., maximal brain conductivity exceeding that of CSF), and was thus replaced by approaches that globally or locally scale the anisotropic conductivities to match the conductivity values available in the literature (Güllmar et al., 2010;Rullmann et al., 2009). Thus, also simulations informed by DTI data suffer from the same uncertainty with regards to the ohmic tissue conductivities as simulations employing isotropic tissues.

In summary, modeling of the brain conductivity as isotropic or anisotropic has only a small to moderate effect on the**E**-fields induced by tES in gray matter and the corresponding simulated current-induced B_z_-fields. Using anisotropic conductivities also does not improve the fit to measured B_z_-fields. On the other hand, as anisotropic brain conductivities estimated from DTI data suffer from the limitations outlined above, it seems unclear whether using them, indeed, helps to improve model accuracy.

### 4.3 Reconstruction of tissue conductivity using DT-MREIT

The DT-MREIT algorithm (Kwon et al., 2014) aims to resolve the problem of uncertain tissue conductivities by employing MREIT data for the estimation of voxel-wise scaling factors for the conversion between diffusion and conductivity tensors. Confirming the validation results in the original study (Kwon et al., 2014), the algorithm performed well for the simulated phantom in our case. For the tested human head model, it achieved good overall reconstruction performance in case of noise-free and complete input data. It can be assumed that it will also work well, for example, for the human extremities that vary slowly along their lengths and can be orientated along the main magnetic field of the scanner.

However, our results also indicate that the current version of the algorithm is less suited for recovering the conductivities of the human head. This has several reasons: First, the DT-MREIT algorithm needs to be informed by the results of the projected current density algorithm (Eroğlu et al., 2021;Jeong et al., 2014). The latter relies on the assumption that the z-component of the current flow is small and can be neglected in order to enable reconstruction of the current density distribution from the MREIT data. This assumption is not fulfilled in case of the human brain. In addition, the signal-to-noise ratio of in-vivo MREIT data from the human brain is limited and further decreases the amount of detail in the reconstructed current density image (Göksu et al., 2021). The accuracy of the DT-MREIT results further depend on the chosen regularization parameter and scaling factor between the diffusion and conductivity tensors at the region boundary. These were systematically optimized in our simulation study to reach the best performance for each case. In practice, this choice is more difficult without access to ground-truth data. These limitations led us to restrict this analysis of the DT-MREIT algorithm to simulations in sub-study 3. It is worth noting that the limitations also apply to the results of a prior study that employed the DT-MREIT algorithm to estimate brain conductivity from in-vivo MREIT data (Chauhan et al., 2018). Finally, the algorithm only recovers the conductivity in regions that provide sufficient MR signal and are inside the imaging field of view. However, the tES-induced electric field in the brain also depends on other regions such as the skull and scalp. Overall, our results suggest that the DT-MREIT algorithm has good reconstruction performance in simplified and ideal situations, such as phantoms that are uniform in the z-direction or noise-free and complete input data of the current flow in the human head. However, its accuracy significantly declines in more realistic scenarios of incomplete input data for complex anatomical regions like the human brain. Principled approaches to set the regularization parameter and scaling factor in these cases are also still lacking. Future work could specifically focus on improving the methods for voxel-wise current density reconstruction, as they appear as the main cause that limits the usefulness of the DT-MREIT algorithm in practice.

### 4.4 Limitations and future direction

Our study focused on the electric field in gray matter as a likely site of the physiological tES effects. The activation of white matter fibers might contribute to the physiological effects of supra-threshold stimulation methods such as transcranial magnetic stimulation or electroconvulsive therapy, and considering anisotropic brain conductivities in corresponding simulations could be more relevant (Lee et al., 2012;Opitz et al., 2011). In addition, the limited resolution of our DTI data causes spatial averaging between cortical layers that might decrease the fractional anisotropy estimates, making gray matter largely isotropic. In high-resolution preclinical and ex-vivo human DTI data (Assaf, 2019), increased anisotropy is observed at a more fine-grained level within some gray matter regions that might locally influence the simulated electric fields. As such, our results might underestimate the impact of anisotropy on the electric fields on the microscopic level. On a related note, the normal component of the**E**-fields in gray matter were slightly more affected by the anisotropic conductivities compared to the**E**-field magnitude. However, these effects remained far lower than the overall uncertainty of the**E**-fields. In addition, we suggest that the above-mentioned limitations of DTI-based conductivity estimates make it difficult to ensure reliable results, in particular regarding the field direction. On the group level, our comparisons were based on metrics that represent average differences or average uncertainties across the GM surfaces. As such, the peak**E**-field differences between simulations based on isotropic versus anisotropic brain conductivities are higher at some cortical positions. However, this does not affect the main finding that these differences are still moderate compared to the general uncertainty of the fields.

The present work focuses on the dependence of the simulated tES-induced electric and magnetic fields on the chosen tissue conductivities. It can be assumed that anatomical inaccuracies of the modeled head volume conductor also contribute to uncertainties in the field estimates (Puonti, Saturnino, et al., 2020), and should be considered in future studies. Specifically, we suggest that the remaining differences between the MREIT data and the simulated magnetic fields after conductivity optimization indicate limitations in the anatomical modeling, including small segmentation errors, subtle segmentation biases (see figure 9 ofPuonti, Saturnino, et al., 2020for an example), and simplifications caused by lumping several tissues into a common class (e.g., modeling skin, fat and muscle as “scalp”). This is supported by the notion that the simulated fields fit generally better to the measurements for the A-P montages compared to the R-L montages. In addition, measurement noise in the MRCDI data adds to the relative errors. In a previous study, we used reference measurements without current injection to show that measurement noise corresponded to relative error levels from 30% to 50% (figure 5D inEroğlu et al., 2021) for the same MR sequence as used here. While we do not have measurements without current injection available for the current dataset, the prior data gives important context information for the interpretation of the results ofFigure 5C.

Here, the conductivity optimization was on purpose performed for each MREIT measurement separately to make the analysis as sensitive as possible to the effects of isotropically versus anisotropically modeled brain conductivities. This choice increases the likelihood of overfitting, so that the optimized conductivities are not necessarily trustworthy. Future studies might focus on combining MREIT data across more subjects and montages, which, together with new MREIT measurement approaches with improved brain coverage, ensures the robustness and generalizability of the results and has the potential to significantly reduce the uncertainty of the tES electric field simulations (Göksu et al., 2023;Gregersen et al., 2024).

In addition, in this study, we focused on healthy subjects to establish a baseline understanding of the effects of brain conductivity on the**E**-field induced by tES. In the future, applying MREIT to patients with neurological disorders, such as stroke, could offer important insights into how these conditions may alter the**E**-field distributions and potentially affect the therapeutic effects. Also, another limitation of our study is the small sample size in sub-study 2, consisting of only five healthy participants. While sub-study 2 confirms the findings of sub-study 1—namely that modeling brain conductivities as anisotropic has only a small to moderate impact on the electric fields compared to other factors—we recognize that a larger sample size would improve the robustness and generalizability of our results. Future studies should thus aim to include a larger and more diverse cohort of participants to validate and extend our findings. Finally, in our study, the amount of conductivity anisotropy (i.e., the ratios between the conductivity eigenvalues) is fully determined by the diffusion anisotropy. Future studies could additionally assess the effects when systematically varying the amount of anisotropy. Based on our results, we expect the effect to be less than the differences between isotropic and anisotropic simulations observed here.

## 5 Conclusion

This study compared two potential factors that may reduce model accuracy for simulations of the electric fields of tES induced in gray matter: The effects of modeling brain conductivities as isotropic rather than anisotropic, and the impact of the generally sparse and variable values are reported in the literature for the tissue conductivities at low frequencies.

Including anisotropic brain conductivities estimated from DTI data resulted in only small to moderate effects on the simulated**E**-field magnitudes that changed on average less than 10% compared to simulations with isotropic conductivities (sub-study 1). In addition, simulation results based on anisotropic brain conductivities did not fit better to measured MREIT data (Bz-fields) compared to the results for isotropic conductivities (sub-study 2). This moderate impact needs to be considered together with the practical limitations when estimating conductivities from diffusion tensors. In particular for gray matter, diffusion tensors measured with standard research-grade diffusion MRI suffer from partial volume effects due to a limited spatial resolution, and the methods to map from diffusion to conductivity tensors lack validation. In combination, these factors make it questionable whether the accuracy of simulated tES-induced electric fields in gray matter is, indeed, improved when employing anisotropic brain conductivities.

Our systematic uncertainty analyses confirmed earlier results showing that the general choice of the conductivity values of the major tissue compartments (in particular gray matter, scalp and skull) clearly affected the simulated tES-induced electric field. The variability of the**E**-field magnitudes caused by the general uncertainty about the tissue conductivities on average clearly exceeded 25% (sub-study 1). In addition, the fit between simulated and measured MREIT data could be consistently improved by the optimization of the conductivities of the tissue compartments—irrespective of whether brain conductivities were modeled as isotropic or anisotropic (sub-study 2). This suggests that model accuracy would generally benefit from more precise and accurate data on the tissue conductivities. It also indicates that MREIT might be a helpful tool for investigating this question.

Finally, we assessed the performance of a specific algorithm, termed DT-MREIT, in reconstructing voxel-wise brain tissue conductivities from a combination of current density and DTI data (sub-study 3). The algorithm performed similarly for current densities simulated with isotropic and anisotropic head models, confirming the results of the two other sub-studies. However, compared with its performance for a phantom featuring a simplified geometry, reconstruction accuracy was low in case of a human head anatomy. We identified the lacking accuracy of the method to reconstruct current densities from raw MREIT data, which is a required preparation step to inform the DT-MREIT algorithm, as the root cause. This strongly suggests that the applicability of DT-MREIT to human brain data is so far limited in practice.

## Supplementary Material

Supplementary Material

## Data Availability

Most of the methods are already publicly available via our open-source software SimNIBS (www.simnibs.org). The scripts for reconstructing the magnetic field measurements from the MR data and conductivity optimization will be made available in a future version of SimNIBS. Until then, they can be obtained from the corresponding author upon reasonable request. The MR data of sub-study 1 and 2 cannot be made available due to privacy restrictions. The scripts and data of sub-study 3 can be downloaded athttps://osf.io/gcu5b/(DOI: 10.17605/OSF.IO/GCU5B).
