# Supplementary material for: The effect of brain tissue anisotropy on the electric field caused by transcranial electric stimulation: Sensitivity analysis and magnetic resonance electrical impedance tomography
Source: Imaging Neurosci (Camb). 2025 Feb 26;3:imag_a_00481. doi: 10.1162/imag_a_00481 (PMC12319980; doi:10.1162/imag_a_00481)
Supplement: Supplementary Material [file imag_a_00481-supp.pdf]

The effect of brain tissue anisotropy on the electric field caused by transcranial electric stimulation: Sensitivity analysis and magnetic resonance electrical impedance tomography

Mohsen Mosayebi-Samani, Teresa Cunha, Hasan Hüseyin Eroğlu, Hartwig Roman Siebner, Michael A. Nitsche, Axel Thielscher

Supplementary Material A: Figures

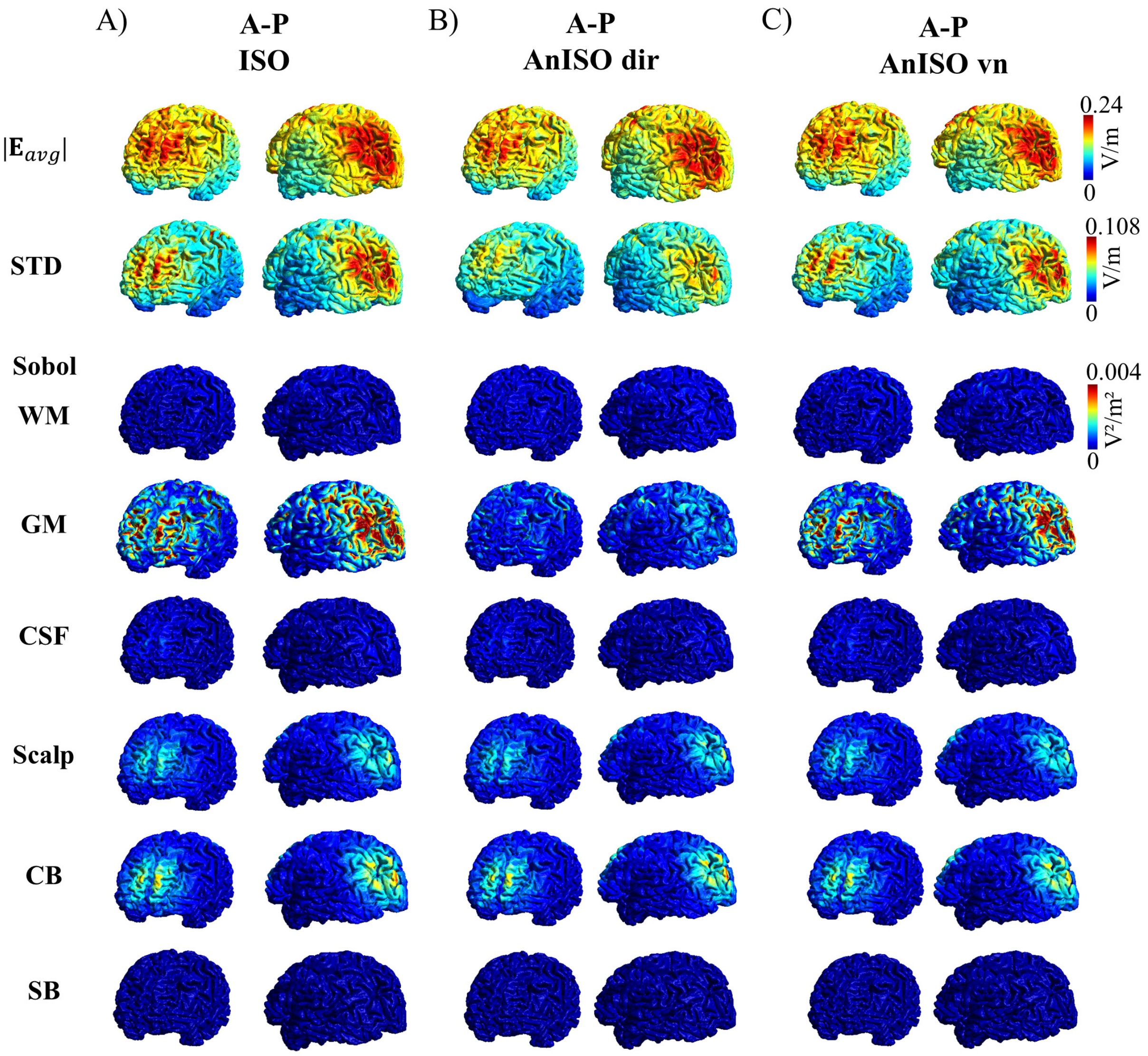

Figure S1. Simulation results of the electric field magnitude on the middle GM surface for the A-P montage (participant 1). The gPC method was used to evaluate the electric field

as a function of conductivities in three different cases: A) Isotropic conductivities (ISO), B) anisotropic brain conductivities based on the 'direct mapping' method (AnISO dir) and 3) anisotropic brain conductivities according to the 'volume normalized' method (AnISO vn). Rows 1 and 2 show the expected average ($|\mathbf{E}_{avg}|$) and standard deviation (STD) of the electric field strength for the uncertainty ranges of the conductivities defined in Table 1 of the main manuscript. Rows 3 to 8 show Sobol indices associated with GM (gray matter), WM (white matter), CSF (cerebrospinal fluid), CB (compact bone) and SP (spongy bone).

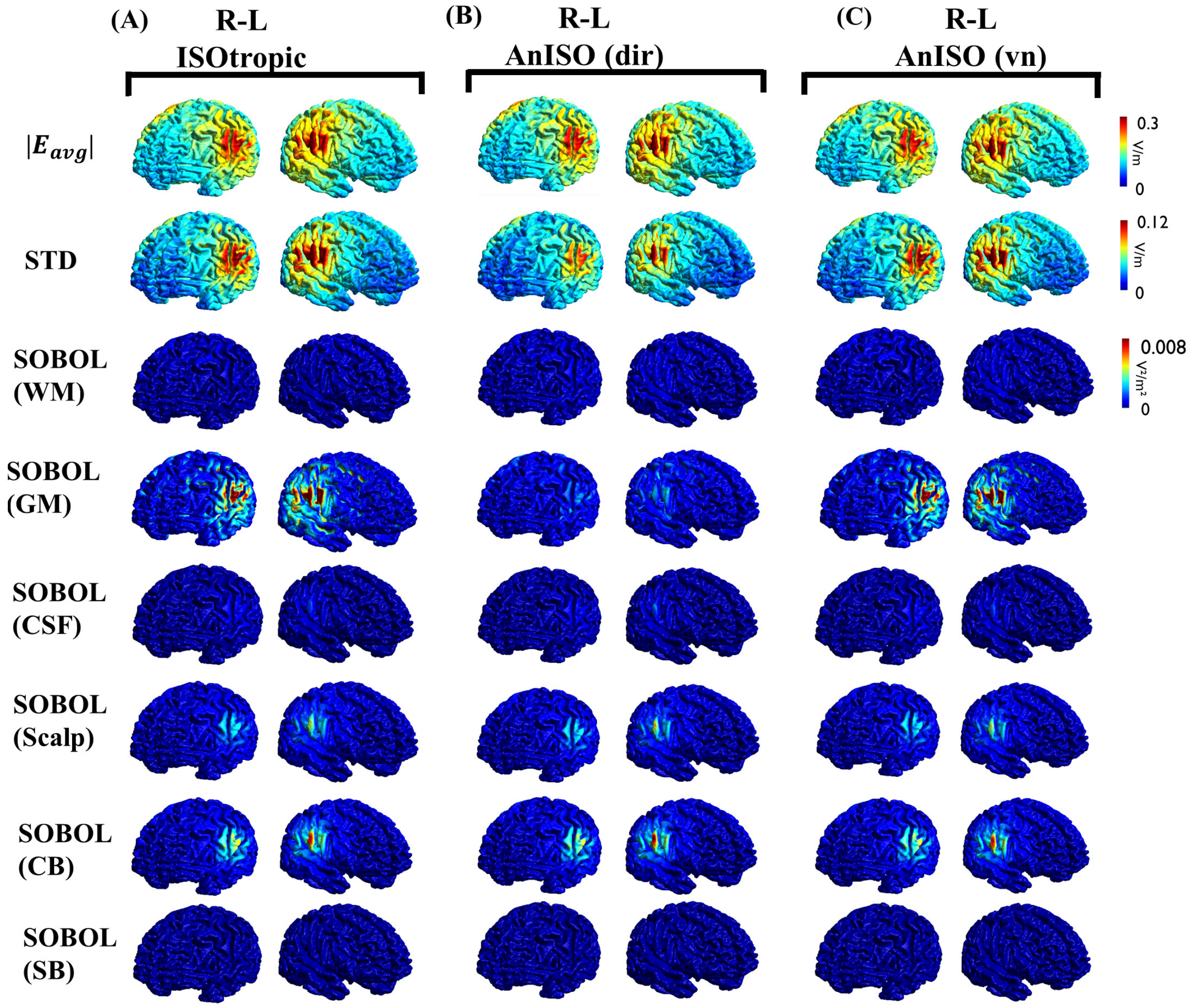

Figure S2. Simulation results of the electric field magnitude on the middle GM surface for the R-L montage (participant 1). The gPC method was used to evaluate the E-fields as a function of conductivities in three different cases of: A) isotropic conductivities, B) anisotropic conductivities based on the 'direct mapping' method (dir) and 3) anisotropic conductivities according to the 'volume normalized' method (vn). The results show the average and standard deviation (STD) of the electric field strength |E| on the middle GM surface. Sobol indices associated with each tissue type. GM: gray matter, WM: White matter, CSF: cerebrospinal fluid, CB: compact bone, SP: spongy bone.

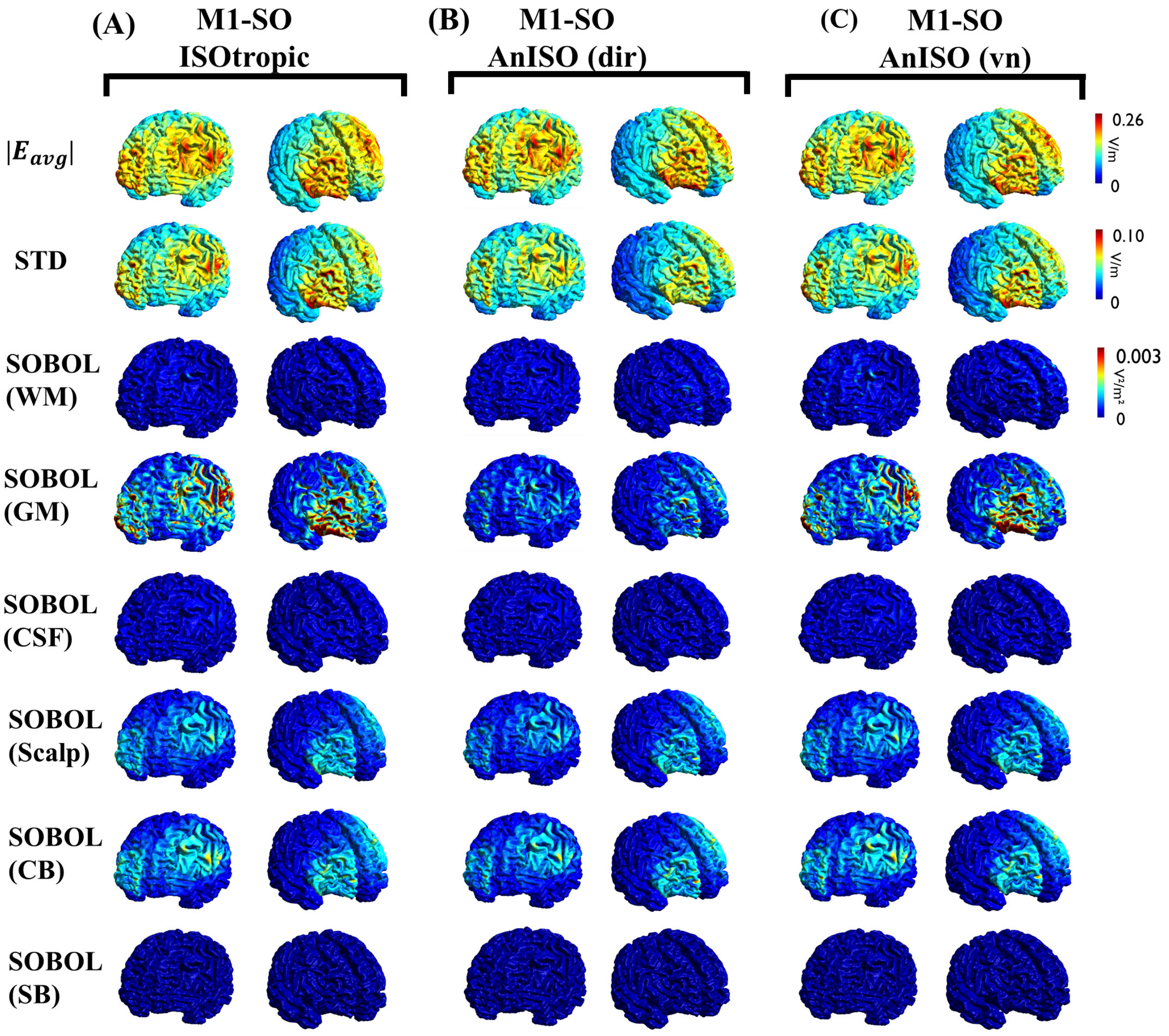

Figure S3. Simulation results of the electric field magnitude on the middle GM surface for the M1-SO montage (participant 1). The gPC method was used to evaluate the E-fields as a function of conductivities in three different cases of: A) isotropic conductivities, B) anisotropic conductivities based on the 'direct mapping' method (dir) and 3) anisotropic conductivities according to the 'volume normalized' method (vn). The results show the average and standard deviation (STD) of the electric field strength |E| on the middle GM surface. Sobol indices associated with each tissue type. GM: gray matter, WM: White matter, CSF: cerebrospinal fluid, CB: compact bone, SP: spongy bone.

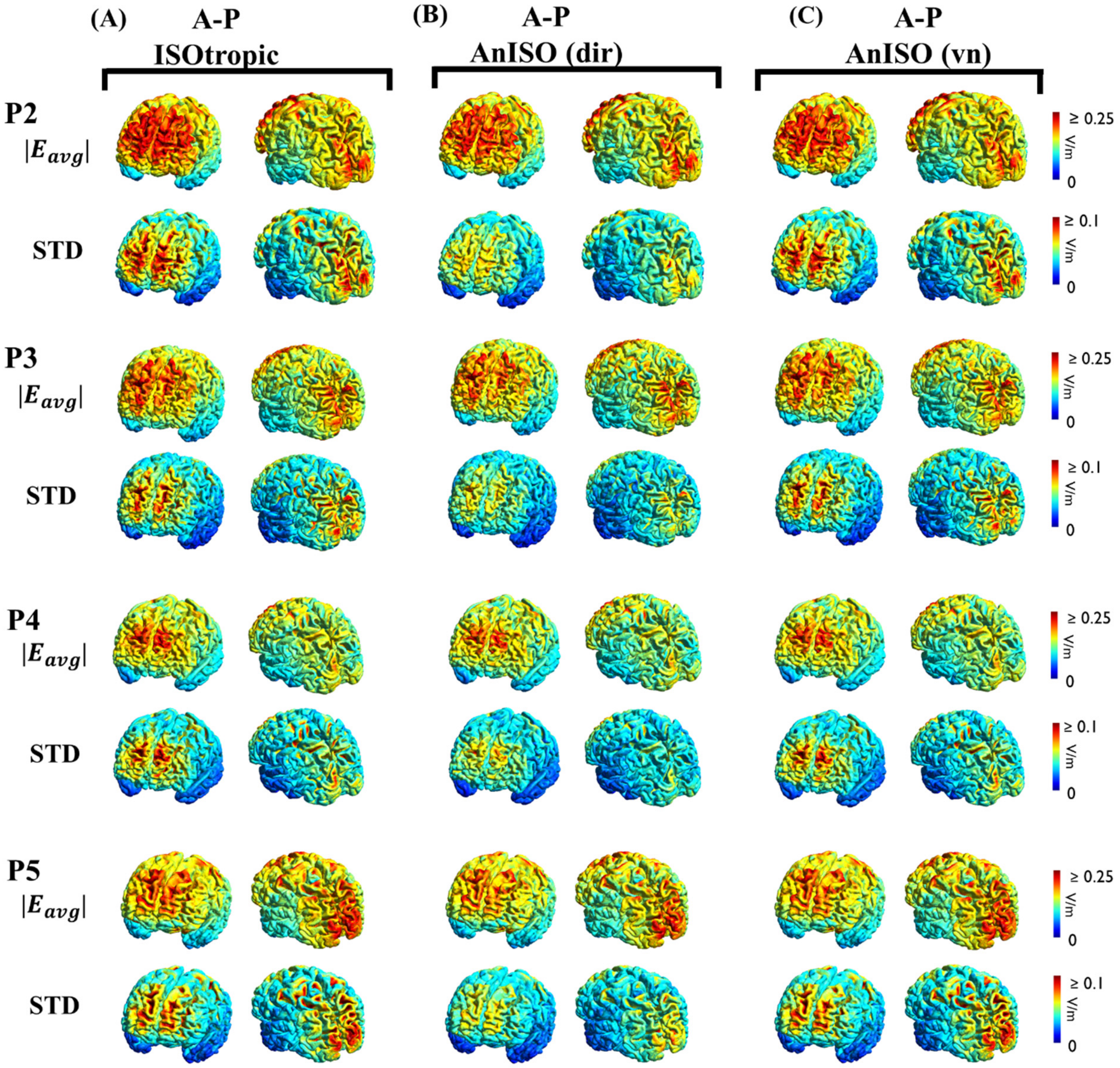

Figure S4. Simulation results of the electric field magnitude on the middle GM surface for A-P montage (participants 2-5). The gPC method was used to evaluate the E-fields as a function of conductivities in three different cases of A) isotropic conductivities, B) anisotropic conductivities based on the 'direct mapping' method (dir) and 3) anisotropic conductivities according to the 'volume normalized' method (vn). The results show the average and standard deviation (STD) of the electric field strength |E| on the middle GM surface.

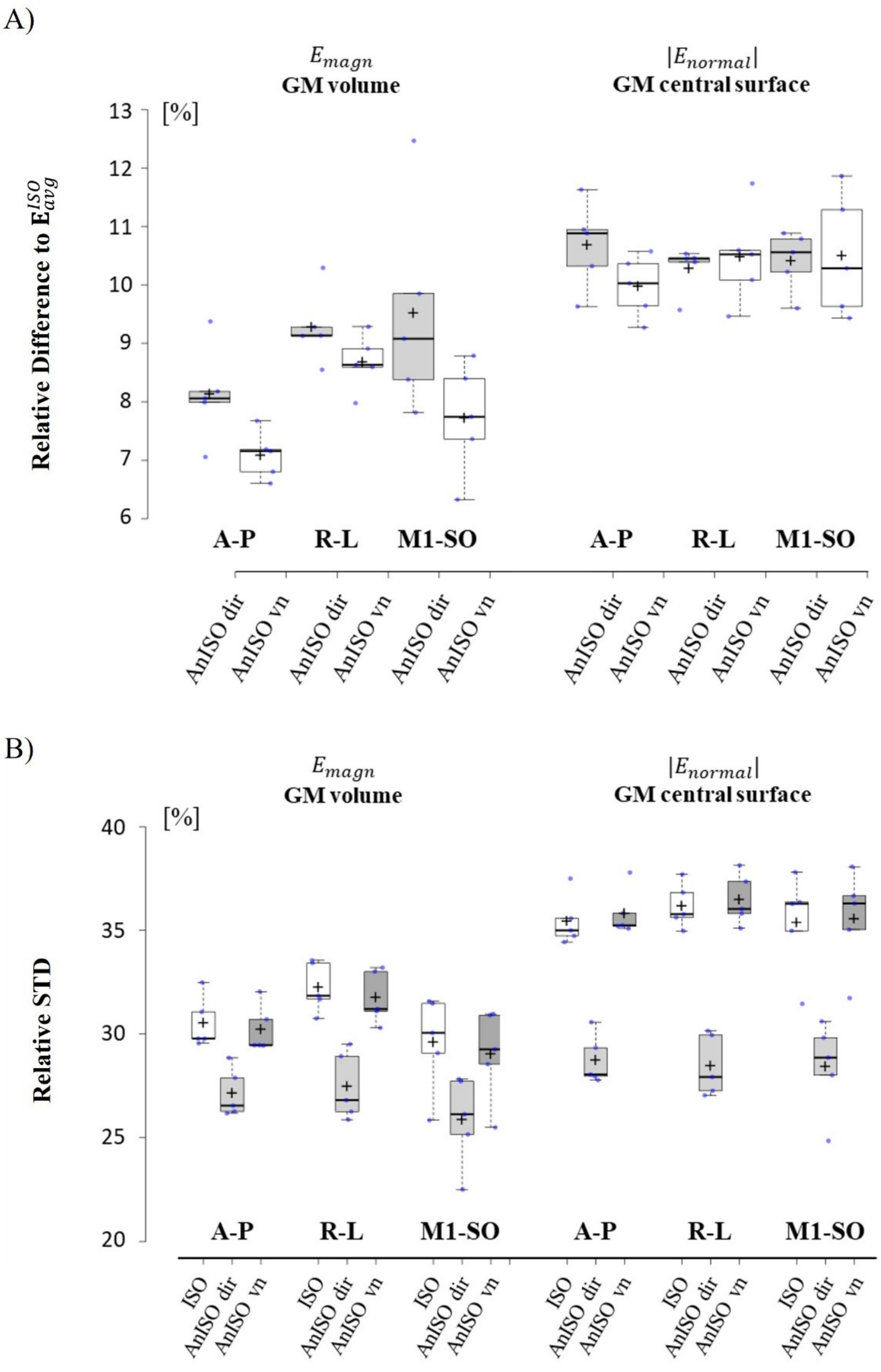

Figure S5. Group results (5 recruited participants) for the simulated electric fields with isotropic and anisotropic conductivities. A) Relative differences between average electric fields, calculated for anisotropic versus isotropic conductivities. B) Relative standard deviations, estimated via the gPC method, of the electric fields. In both subfigures, the leftmost set of differences refers to the electric field magnitudes computed in the Gray Matter (GM) volume, while the rightmost set depicts the normal component of the electric field calculated in the central GM surface. Box plots: Whiskers extend to data points that are the 5th and 95th percentile, together with the mean (+) and median (-) across participants. Individual data points are shown as blue dots.

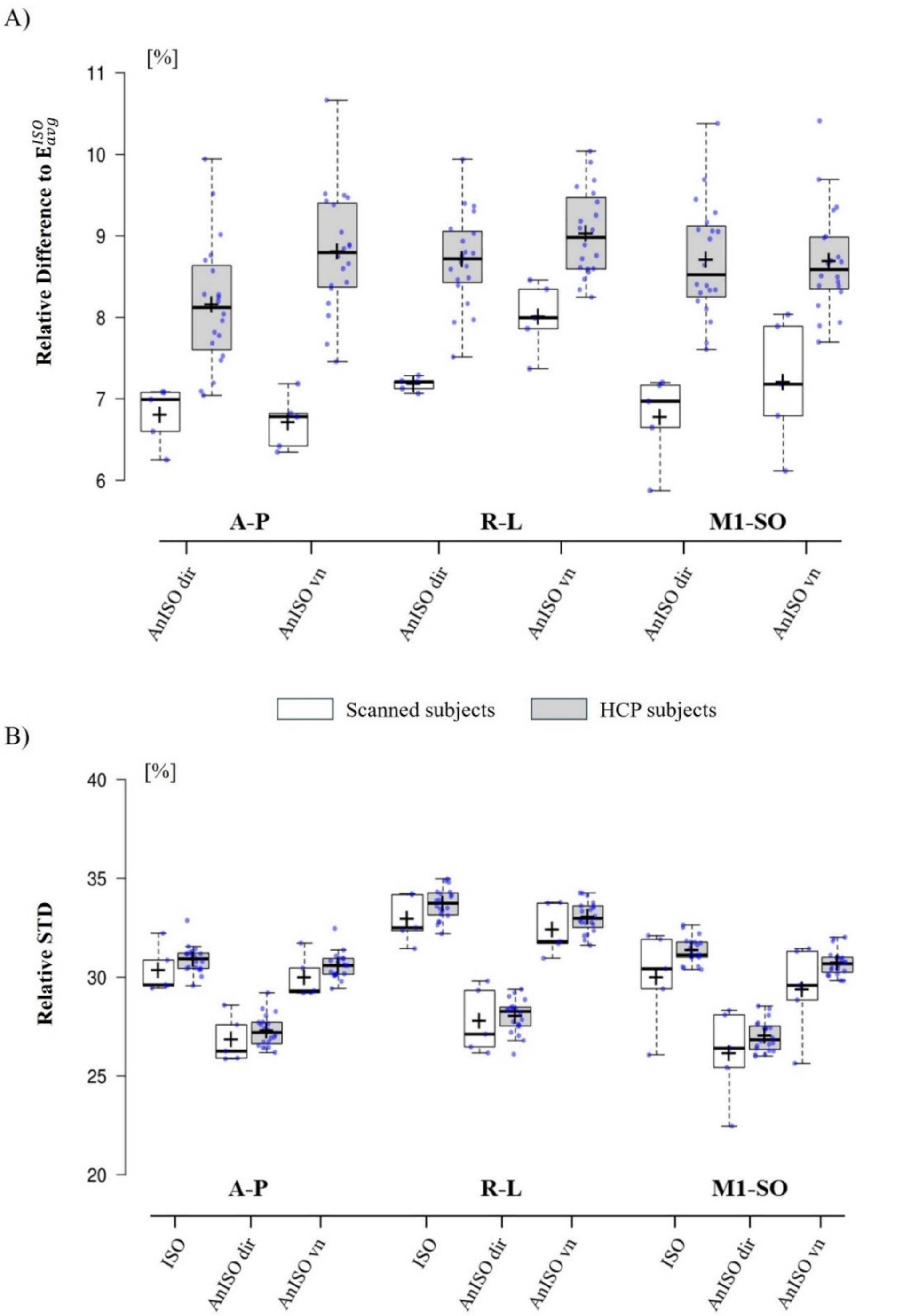

Figure S6. Group results (5 recruited versus 20 HCP participants) for the simulated electric fields with isotropic and anisotropic conductivities. The used HCP data is part of the '1200 Subjects Data Release', whose acquisition details are described in 'Appendix 1: HCP scan protocols. For further details, see (Sotiropoulos et al., 2013). The water diffusion tensors were computed from single-shell diffusion-weighted data (only b=1000 in addition to the b=0 scans). A) Relative differences between average electric field magnitudes calculated in the central GM surface for anisotropic versus isotropic conductivities. B) Relative standard deviations, estimated via the gPC method, of the electric field magnitudes calculated in the central GM surface. Box plots: Whiskers extend to data points that are the 5th and 95th percentile, together with the mean (+) and median (-) across participants. Individual data points are shown as blue dots.

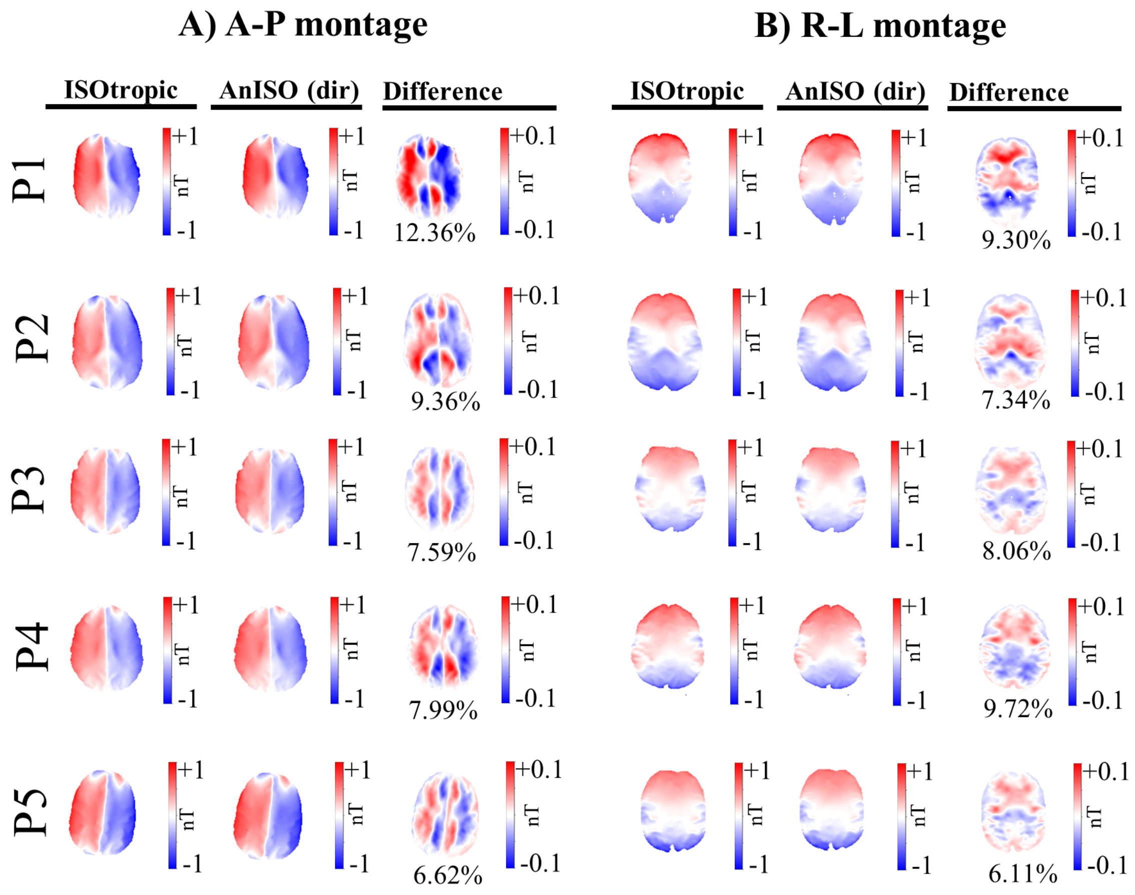

Figure S7. Differences between the simulated B_z-fields with isotropic conductivities and anisotropic conductivities. For each participant (P1-5), the B_z-field distributions within GM, WM and CSF were calculated in horizontal slices for isotropic or anisotropic ('direct mapping' method; AnISO (dir)) conductivities. The absolute differences between both are shown in the third column. In addition, the relative differences (eq. 7 in the main paper) are stated below each slice.

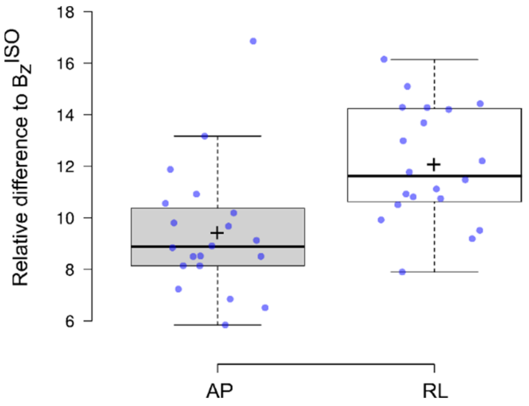

Figure S8. Relative differences between the B_z-fields calculated for anisotropic (AnISO dir) versus isotropic conductivities for the 20 HCP subjects. The Bz-fields were simulated in transversal slices going through or in between the electrode centers, for R-L and A-P montages, respectively. The simulations were based on conductivities taken from the literature (see Table 1 in the main text). Box plots: Whiskers extend to data points that are the 5th and 95th percentile, together with the mean (+) and median (-) across participants. Individual data points are shown as blue dots.

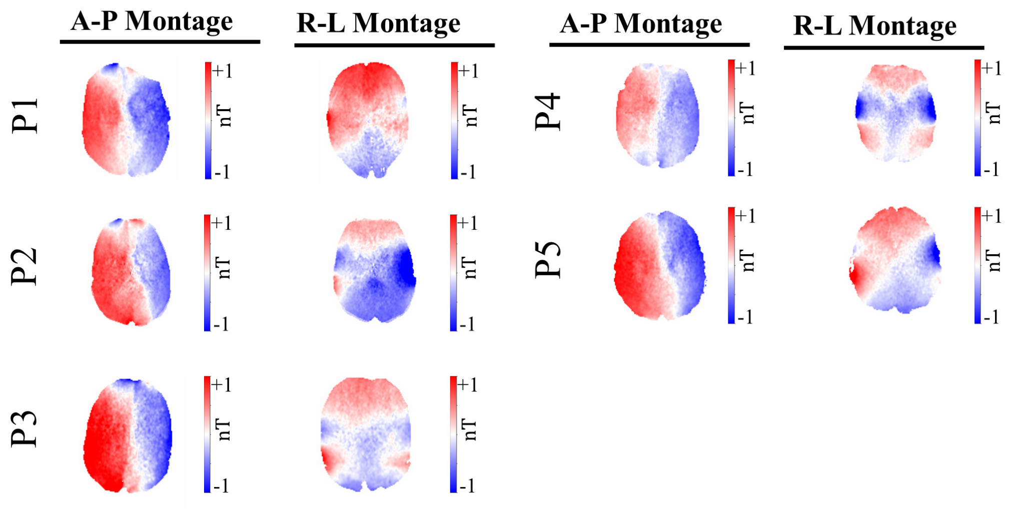

Figure S9. Experimental B_z measurements for 5 participants (P1-5), each with A-P and R-L montages, for a current injection of ±1 mA. For acquisition details, see section 2.2 of the main paper.

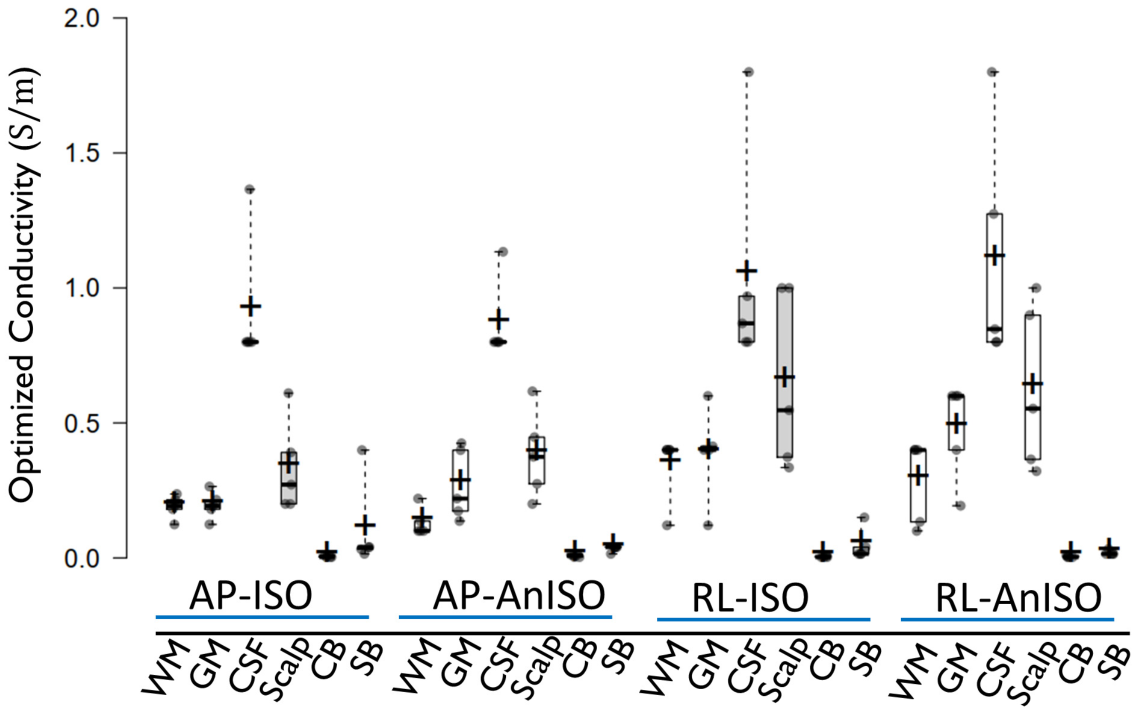

Figure S10. Using the B_z data of the 5 participants (each with A-P and R-L montages) as B_z^{ref}, we applied the optimization algorithm (eq. 7 of the main manuscript) for 1) isotropic and homogeneous model conductivities (ISO), and 2) anisotropic GM and WM conductivities according to the 'direct mapping' method (AnISO).

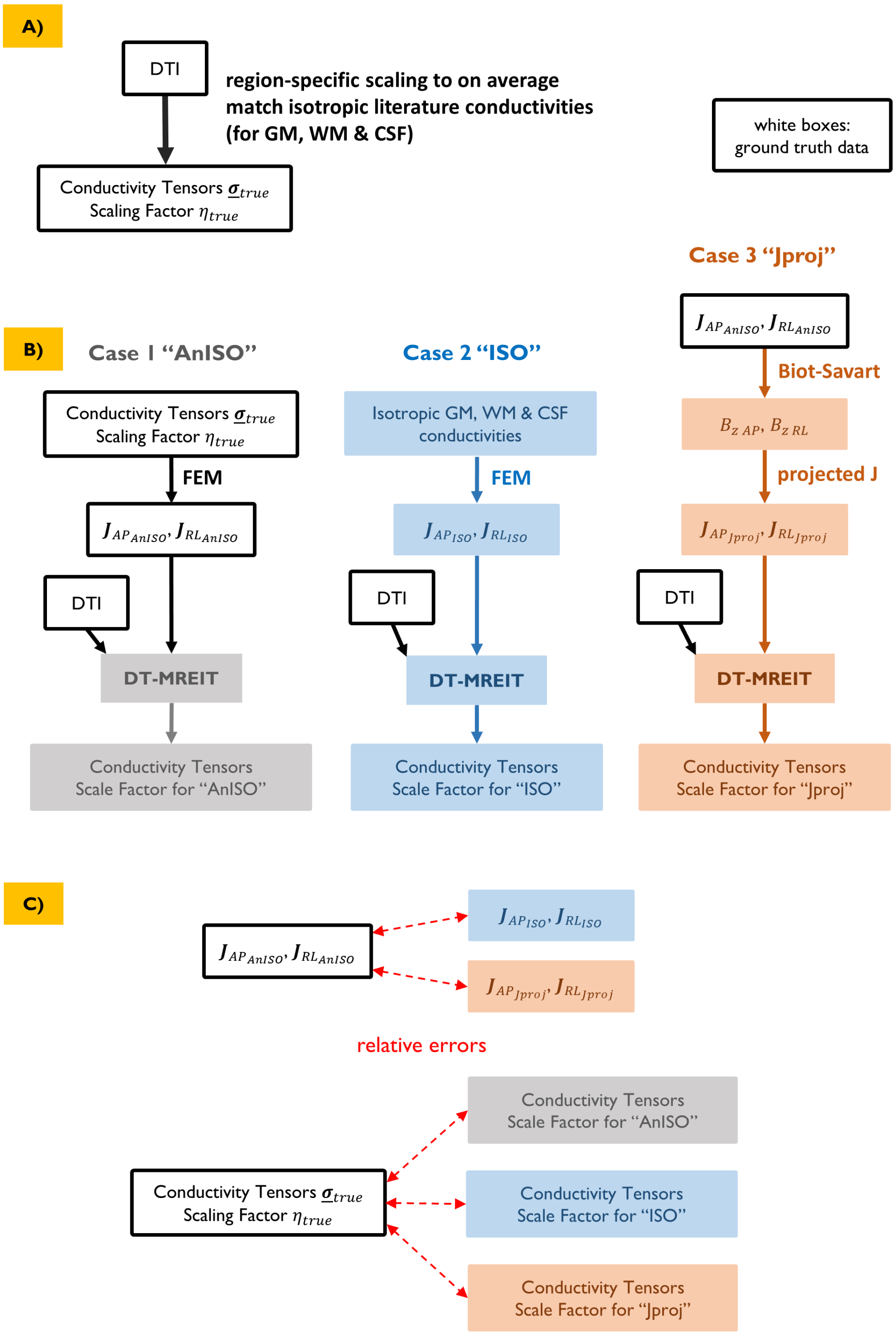

Figure S11. A) Creation of ground-truth conductivity tensors: The DTI tensors are rescaled (using separate scale factors for GM, WM and CSF) so that their region averages match the isotropic conductivities reported in the literature. B) Using DT-MREIT, conductivity tensors are reconstructed from the DTI and simulated current density data (AP and RL montages) for three different cases. **AnISO**: Current density data simulated using the ground-truth conductivity tensors. **ISO**: Current density data simulated with isotropic literature conductivities. **Jproj**: Current densities recovered from the true current-induced magnetic fields by means of the projected current density algorithm. C) Relative errors of current densities are determined for the ISO and Jproj cases (using the AnISO current densities as ground truth). In addition, relative errors are calculated for the conductivity tensors and scale factors for all three cases compared to the ground-truth conductivity tensors and scale factors.

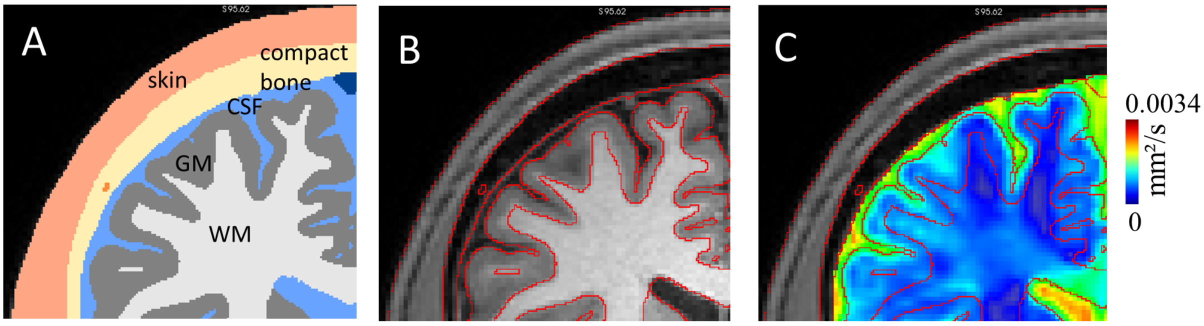

Figure S12. Coronal slice of the 'ernie' head model. A) Tissue segmentation. B) T1-weighted image with boundaries of the segmented tissue compartments shown in red. C) First component of the diffusion tensor included in the dataset, prepared using the SimNIBS script *dwi2cond*. The diffusion tensor images were coregistered to the T1-weighted image and upsampled to 1 mm^3 isotropic resolution from their original resolution of 1.9x1.9x2.0 mm^3. The partial volume effects are clearly visible as increased diffusion values (light blue) within GM at the boundary towards CSF.

Supplementary Material B:

As additional control analysis, we evaluated the combined effects of the ‘direct mapping’ method and the gPC method on the conductivity ranges for GM and WM. For assessing the uncertainty of the electric field in GM via gPC, the GM and WM conductivities are independently sampled from their respective probability density functions (PDFs), and then combined into a common scaling factor when using the ‘direct mapping’ method (see eq. 2 of the main paper). As a result, the scaling factor has lower variability compared to each of the GM and WM conductivities alone. However, ‘direct mapping’ also introduces spatial inhomogeneity of conductivities (due to spatial variability of the mean diffusivity of the DTI data), which is absent for isotropic conductivities and the ‘volume normalized’ mapping. For ‘direct mapping’, these two effects in combination will determine the overall range of conductivity values that occur within the GM and WM compartments. The following analysis confirms that this overall range remained similar to the intended conductivity ranges for GM and WM, as given in Table 1 of the main paper.

In total, N=1000 samples of gray matter ($0.1<\sigma\_GM<0.6$) and white matter ($0.1<\sigma\_WM<0.4$) conductivities were drawn from their PDFs and used together with the DTI data to calculate GM and WM conductivity tensors using the ‘direct mapping’ method (eq. 1-3 in the main paper). The minimum and maximum eigenvalues were determined separately for GM and WM for each of the 1000 samples. Subsequently, histograms of the resulting values were constructed.

The histograms in Fig. S13 depict the distributions of the minimum and maximum conductivity eigenvalues of gray matter (Fig. S13A) and white matter (Fig. S13B) voxels. They indicate that ‘direct mapping’ maintains similar levels of variability for the GM and WM as intended (Table 1 of the main paper), even though it tends to increase the variability in WM while reducing it in GM.

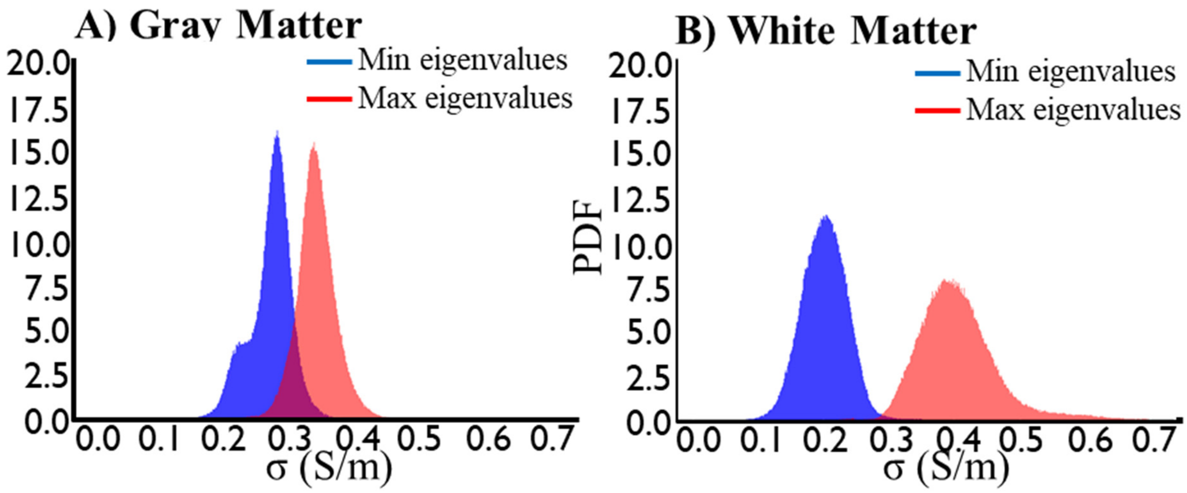

Figure S13. Distribution of conductivity eigenvalues using the 'direct mapping' method. We sampled 1000 GM ($0.1 < \sigma\_GM < 0.6$) and WM ($0.1 < \sigma\_WM < 0.4$) conductivities from their respective PDFs (used for gPC model) and calculated the GM and WM conductivity tensors. The histograms show the distribution of minimum and maximum conductivity eigenvalues for GM (A) and WM (B) voxels, confirming that the 'direct mapping' method maintains the intended conductivity ranges for GM and WM as specified in Table 1 of the main paper.

Supplementary Material C: DT-MREIT for phantom data

Volume conductor model

We constructed a computational model of a three-dimensional cylindrical phantom similar to that used in (Kwon et al., 2014) (Fig. S11A). The phantom includes two pairs of attached electrodes on its surface to inject currents (for A-P and R-L current injection), and four additional objects: One isotropic cylinder object (agar gel) and three anisotropic objects (muscle tissue) with different directional diffusion tensors **D** (Table S1). The background of the model was set to a constant isotropic conductivity. Table S1 includes the conductivities of the phantom elements and the used parameters to generate the diffusion tensor map **D** (Fig. S11B). The directional diffusion tensors are set similar to (Kwon et al., 2014) using the equation

$$\mathbf{D} = \begin{pmatrix} \cos\alpha & \sin\alpha & 0 \\ -\sin\alpha & \cos\alpha & 0 \\ 0 & 0 & 1 \end{pmatrix} * \begin{pmatrix} d_1 & 0 & 0 \\ 0 & d_2 & 0 \\ 0 & 0 & d_3 \end{pmatrix} * \begin{pmatrix} \cos\alpha & \sin\alpha & 0 \\ -\sin\alpha & \cos\alpha & 0 \\ 0 & 0 & 1 \end{pmatrix}^T \quad \text{(eq. S1)}$$

where α is the orientation specified in Table S1. Conductivity tensors (Fig. S11C) were created by scaling the DTI data of each region so that the geometric means of the conductivity eigenvalues matched the isotropic conductivities listed in Table S1.

For comparisons, we also created a version of the phantom model with isotropic and homogenous conductivities within each region, using the conductivities shown in Table S1.

Simulations

Please refer to “2.7.3. Simulation details” of the main manuscript for details.

Results

The current densities in the phantom model (top rows in Figs. S11D-F) are very similar for all three cases and the J_z component is consistently very low (relative errors of $|\boldsymbol{J}|$ for *ISO* vs *AnISO:* AP 4.7% and RL 2.9%; *Jproj* vs *AnISO:* AP 7.7% and RL 6.2%). This results in a relatively good match between the geometrical means of the ground-truth conductivity tensors used for the current-flow simulations and the respective conductivity tensors reconstructed by DT-MREIT (bottom rows in Figs. S11D-F; relative errors for *AnISO*, *ISO* and *Jproj*: 18.8%, 19.3% and 21.6%; Table S2 lists the region-wise averages of the reconstructed conductivities). Also the relative error for the scale factors remains low for all three cases (*AnISO*, *ISO* and *Jproj*: 15.2%, 16.2% & 16.8%).

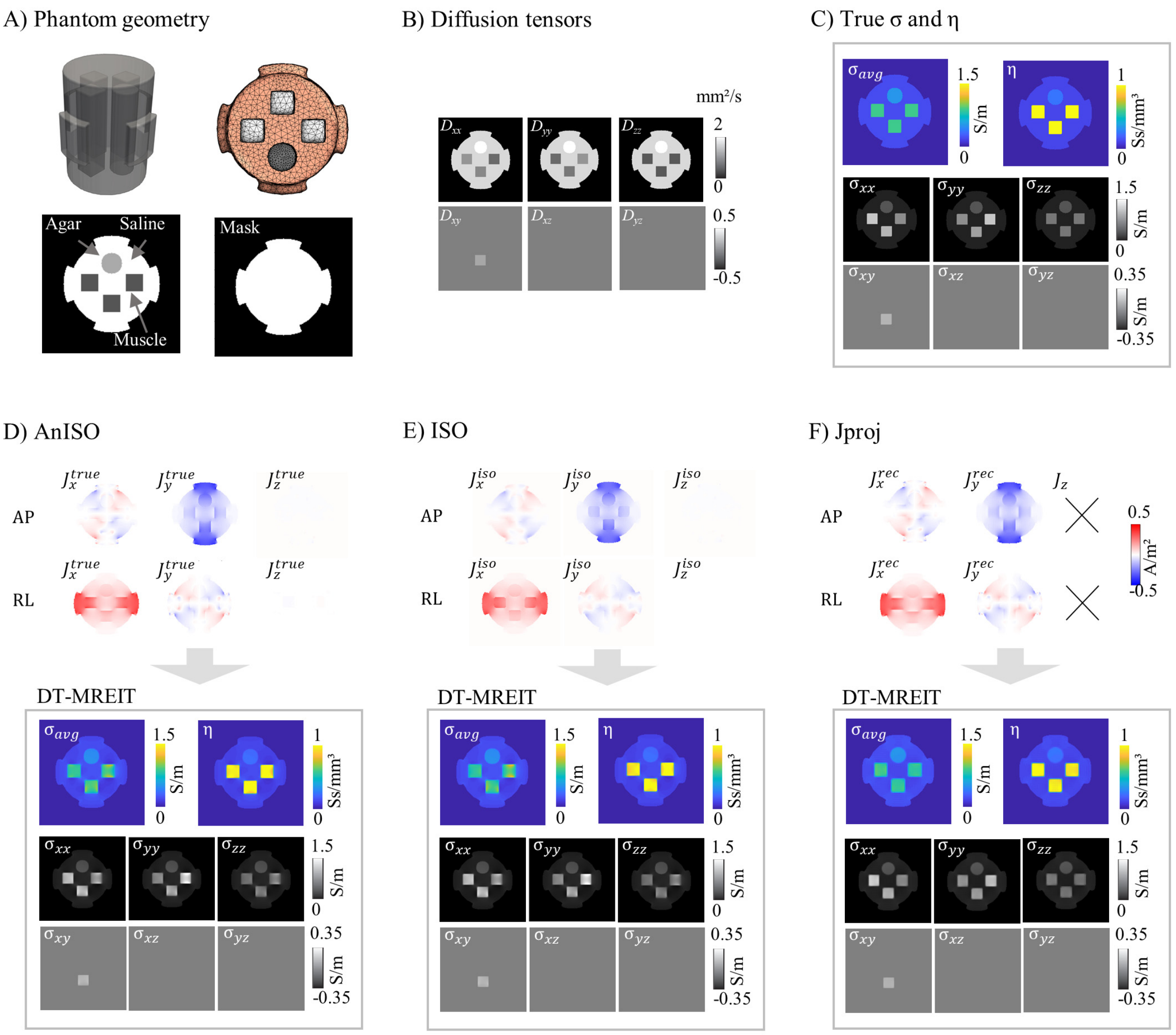

Figure S14. DT-MREIT results (Phantom model). A) Three-dimensional cylindrical phantom model, with four recessions for attaching two pairs of electrodes (1mA baseline-to-peak; montage 1: A-P; montage 2: R-L). The phantom includes four objects embedded in an isotropic background (saline): One cylinder containing isotropic material (agar gel) and three rectangular objects with anisotropic material (muscle tissue) with different directional diffusion tensors D (eq. S1 and Table S1). B) Components of the diffusion tensors D in a horizontal slice, used as input to the DT-MREIT algorithm. C) Ground-truth conductivities σ and scale factors η (σavg denote the geometric mean of the eigenvalues of the conductivity tensors, σ_{xx}, σ_{yy}, etc. indicate the different tensor components). D) **"AnISO"**: Simulated current density distributions for anisotropic (ground-truth) conductivities (top rows) and reconstructed conductivities and scale factors (bottom rows). E) **"ISO"**: Simulated current density distributions for matched isotropic conductivities (top rows) and reconstructed conductivities and scale factors (bottom rows). F) **"Jproj"**: Current densities determined with the projected current density algorithm (eq. 8 of the main paper) (top rows) and reconstructed conductivities and scale factors (bottom rows) are shown.

Table S1. The conductivities of the objects included in the phantom and the parameters used to generate the diffusion tensor map. d_i are diffusion eigenvalues, σ_{iso} is the isotropic conductivity and α is the orientation used for the diffusion tensors inside the objects (eq. S1). The phantom parameters are selected according to (Kwon et al., 2014).

**Parameters/Tissues**	**Saline**	**Agar**	**Muscle**
d_1 (10^{-3} $mm^2 s^{-1}$)	1.70	2.10	1.17
d_2 (10^{-3} $mm^2 s^{-1}$)	1.70	2.10	0.84
d_3 (10^{-3} $mm^2 s^{-1}$)	1.70	2.10	0.70
σ_{iso} [S/m]	0.2	0.5	0.88
α (rad)	0	0	0, 0.6, π/2

Table S2. Geometric means of the eigenvalues of the conductivity tensors in the three tissue types (saline, agar, muscle) of the phantom. Listed are the averages ± standard deviation within each tissue (units: S/m).

**Tissue**	**Ground truth**	**AnISO**	**ISO**	**Jproj**
**Saline**	0.2 ± 0.0	0.19 ± 0.07	0.19 ± 0.06	0.21 ± 0.08
**Agar**	0.5 ± 0.0	0.46 ± 0.03	0.46 ± 0.03	0.48 ± 0.03
**Muscle**	0.88 ± 0.0	0.86 ± 0.11	0.85 ± 0.12	0.81 ± 0.10

REFERENCES

Kwon, O. I., Jeong, W. C., Sajib, S. Z., Kim, H. J., & Woo, E. J. (2014). Anisotropic conductivity tensor imaging in MREIT using directional diffusion rate of water molecules. *Phys Med Biol*, *59*(12), 2955–2974. https://doi.org/10.1088/0031-9155/59/12/2955

Sotiropoulos, S. N., Moeller, S., Jbabdi, S., Xu, J., Andersson, J. L., Auerbach, E. J., Yacoub, E., Feinberg, D., Setsompop, K., & Wald, L. L. (2013). Effects of image reconstruction on fiber orientation mapping from multichannel diffusion MRI: reducing the noise floor using SENSE. *Magnetic Resonance in Medicine*, *70*(6), 1682–1689.
